# Insights gained from a comprehensive all-against-all transcription factor binding motif benchmarking study

**DOI:** 10.1186/s13059-020-01996-3

**Published:** 2020-05-11

**Authors:** Giovanna Ambrosini, Ilya Vorontsov, Dmitry Penzar, Romain Groux, Oriol Fornes, Daria D. Nikolaeva, Benoit Ballester, Jan Grau, Ivo Grosse, Vsevolod Makeev, Ivan Kulakovskiy, Philipp Bucher

**Affiliations:** 1 School of Life Sciences, Ecole Polytechnique Fédérale de Lausanne (EPFL), CH-1015 Lausanne, Switzerland; 2 Swiss Institute of Bioinformatics (SIB), CH-1015 Lausanne, Switzerland; 3 Vavilov Institute of General Genetics, Russian Academy of Sciences, Gubkina 3, Moscow, Russia 119991; 4 Institute of Protein Research, Russian Academy of Sciences, Institutskaya 4, Pushchino, Russia 142290; 5 Faculty of Bioengineering and Bioinformatics, Lomonosov Moscow State University, Leninskiye gory 1-73, Moscow, Russia 119234; 6 Moscow Institute of Physics and Technology (State University), Institutskiy per. 9, Dolgoprudny, Russia 141700; 7 Centre for Molecular Medicine and Therapeutics, Department of Medical Genetics, BC Children’s Hospital Research Institute, University of British Columbia, Vancouver, BC V5Z 4H4 Canada; 8 Aix Marseille Université, INSERM, TAGC, Marseille, France; 9 Institute of Computer Science, Martin Luther University Halle-Wittenberg, Halle (Saale), Germany; 10 German Centre for Integrative Biodiversity Research (iDiv) Halle-Jena-Leipzig, Leipzig, Germany; 11 Engelhardt Institute of Molecular Biology, Russian Academy of Sciences, Vavilova 32, Moscow, Russia 119991

**Keywords:** PWM, Transcription factor binding sites, Benchmarking, ChIP-seq, HT-SELEX, PBM

## Abstract

**Background:**

Positional weight matrix (PWM) is a de facto standard model to describe transcription factor (TF) DNA binding specificities. PWMs inferred from in vivo or in vitro data are stored in many databases and used in a plethora of biological applications. This calls for comprehensive benchmarking of public PWM models with large experimental reference sets.

**Results:**

Here we report results from all-against-all benchmarking of PWM models for DNA binding sites of human TFs on a large compilation of in vitro (HT-SELEX, PBM) and in vivo (ChIP-seq) binding data. We observe that the best performing PWM for a given TF often belongs to another TF, usually from the same family. Occasionally, binding specificity is correlated with the structural class of the DNA binding domain, indicated by good cross-family performance measures. Benchmarking-based selection of family-representative motifs is more effective than motif clustering-based approaches. Overall, there is good agreement between in vitro and in vivo performance measures. However, for some in vivo experiments, the best performing PWM is assigned to an unrelated TF, indicating a binding mode involving protein-protein cooperativity.

**Conclusions:**

In an all-against-all setting, we compute more than 18 million performance measure values for different PWM-experiment combinations and offer these results as a public resource to the research community. The benchmarking protocols are provided via a web interface and as docker images. The methods and results from this study may help others make better use of public TF specificity models, as well as public TF binding data sets.

## Background

The system of gene regulation in eukaryotes is a complex network of interdependent processes, from epigenetic marking of various chromatin states to alternative splicing and varying translation efficiency of particular transcripts. At the very heart of this system lies the process of mRNA transcription, governed by the transcription factor (TF) proteins, recruiting RNA pol II and determining its activity in different regions of the genome by recognizing and binding particular DNA sequences.

**Fig. 1:**
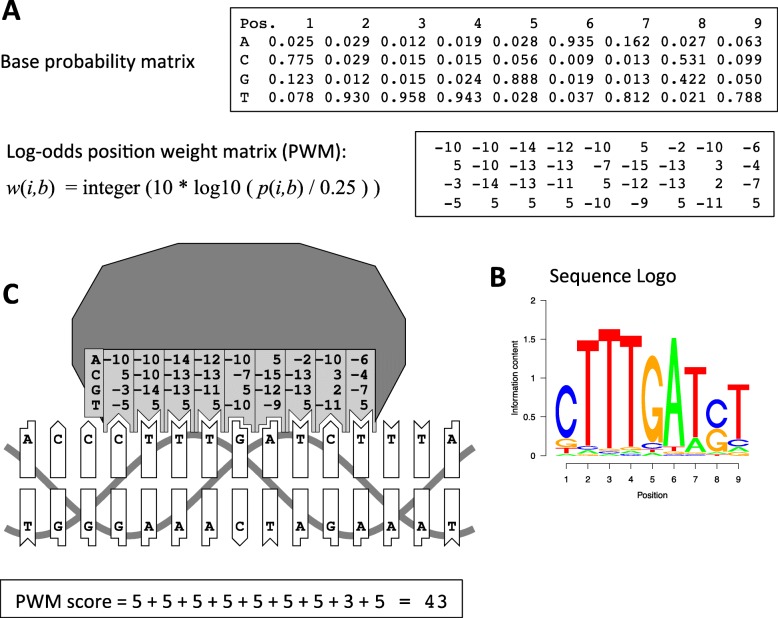
Position weight matrix (PWM) model for representing transcription factor (TFBS) binding site motifs. **a** Base probability and position weight matrices are two alternative representations of a TFBS motif, inter-convertible by the formula shown. **b** Sequence logo representation of the same motif. **c** Biophysical interpretation of the PWM model

TFs recognize short (10–20 bp long) sequence motifs, which are typically described by so-called position-specific weight matrices [1], see Fig. 1. A position weight matrix (PWM) assigns scores to potential target sequences. This score is related to the binding energy of a TF for a particular stretch of nucleotides. The binding mechanism being quantitative rather than qualitative, the purpose of a PWM is not only to identify potential binding sites in a sequence but also to predict their relative strength of binding. Even though the PWM model has been criticized for its simplicity and intrinsic limitations, it is likely to remain the community standard for many years to come, as many popular DNA sequence analysis platforms use it and are unlikely to support a new type of model in the near future.

PWMs come in several forms. The classical PWM representation features numbers for each base at each position of the motif, which are summed up to compute the binding score for a candidate DNA sequence. Base frequency matrices reflect the probabilities at which individual bases occur at respective binding site locations. The two representations are inter-convertible. The sequence motif encoded by a base frequency matrix is often visualized by a so-called sequence logo, in which the combined height of the letters at a particular position corresponds to the Information Content of the underlying base probability distribution, as defined by Shannon’s formula.

A PWM can be interpreted as a biophysical model of a sequence-specific protein-DNA interaction. The columns of the matrix correspond to “base-pair acceptor sites” on the protein surface. A binding score for the bound DNA subsequence is computed by adding up scores for interacting bases along the motif. These scores are supposed to be negatively correlated to the binding free energy of the protein-DNA complex.

PWM-based TF binding motifs are often inferred from data generated with high-throughput assays for genome-wide in vivo binding site mapping such as those based on chromatin immunoprecipitation (ChIP-chip, ChIP-seq, and related methods), e.g., [2], or high-throughput SELEX (HT-SELEX) for in vitro selection of TF binding sequences [3], or protein-binding microarrays (PBM) for quantitative TF affinity measurements of large numbers of double-stranded oligonucleotides [4]. A great variety of computational algorithms for building specificity models from such data have been published, recently reviewed in [5]. Most of these algorithms generate at some stage an alignment of putative binding sites from which a base probability matrix is derived.

### Overview of current TF motif databases

Starting with the first experiments characterizing TF specificity by low-throughput studies, numerous attempts were initiated to aggregate and systematize the resulting data, mostly, by collecting and aligning the sequences of the binding sites, producing the PWM models, and providing them in the form of motif collections. One of the first and most widely known databases was TRANSFAC [6], which probably still remains the largest, although proprietary, collection of PWMs from curated low-throughput data related to mammalian TFs. Open-access databases such as JASPAR [7] had notably less volume until the rise of high-throughput data, which significantly reduced the need for literature mining but put an emphasis on the curation of the PWMs and application of more sophisticated motif discovery tools.

Nowadays, most of the major motif collections, from species-centric (e.g., FlyFactorSurvey [8], YeTFaSCo [9], Plant Cistrome Database [10], HOCOMOCO [11]), or technology-specific (e.g., UniPROBE [12]) to all-inclusive resources (e.g., CIS-BP [13], FootprintDB [14], iRegulon [15], and HOMER [16]), all use one or another form of the PWM as the primary (and most often the only) motif representation.

### Previous benchmarking efforts and evaluation protocols

Early benchmarking protocols for PWMs were developed for the purpose of evaluating motif discovery algorithms, see [17] and [18], focusing on eukaryotic and prokaryotic regulatory regions, respectively. The performance measures were based on a comparison between predicted binding site locations and annotated binding sites from the biological literature (as presented in TRANSFAC). The results were rather sobering leading to the conclusion that motifs discovered from the small training sets available at that time were generally not accurate enough for being useful to biologists. However, the ground truth used in these studies could be criticized. For many reference binding sites, only the existence of a binding site had experimental support (e.g., from a DNase I footprint), whereas the precise boundaries of the site, and the identity of the interacting TF was inferred from published binding site consensus sequences. The evaluation was thus partly circular, which may have resulted in an underestimation of the performance of the newly discovered motif.

New benchmarking methods were introduced in the context of a prediction challenge aimed at benchmarking algorithms for modeling TF binding specificity from high-throughput data, such as those obtained from protein-binding microarrays (PBMs) [19]. Participants were given training data for building a model and then asked to use the model to predict the binding strength of 35-bp-long test sequences. The evaluation was either based on full-length sequence scores or 8-mer enrichment scores derived from the full-length scores. Pearson correlation and AUC ROC (area under the curve of the receiver operating characteristic) were used as performance measures, the latter requiring a threshold-dependent classification of the sequences into binders and non-binders. The progress with regard to the previous method was that these protocols did not rely on motif-inferred binding site locations, thus avoiding the circularity pointed out in the preceding paragraph.

Orenstein and Shamir were to our knowledge the first to propose benchmarking protocols taking as input PWM models directly, rather than predictions made with such models [20]. The purpose of the study was to compare PWM models derived with two in vitro technologies, HT-SELEX and PBM, and to assess their capacity to predict in vivo TF binding sites, using ChIP-seq data as the ground truth. As a central component of their methodology, they proposed a “sum occupancy score,” which serves to compute an inclusive binding score for DNA sequences longer than the PWM under consideration. Based on these scores, they evaluated the PWM’s capacity to discriminate between ChIP-seq peak regions and random genomic sequences, choosing AUC ROC as a performance measure.

The most comprehensive PWM benchmarking study carried out so far was presented by Kibet and Machanick [21] and involved over 6000 TF motifs from 14 different resources. ChIP-seq peaks and PBM measurements were used as the test data. The study compared several scoring functions and performance measures, and newly introduced central motif enrichment in ChIP-seq peak regions, computed with CentriMo [22], as a performance criterion.

### Novelty and objectives of this study

A limitation of all previous studies was that TF binding motifs were only benchmarked against experimental data for the same TF (the TF to which the PWM is assigned in the motif database). This approach ignores the fact that TFs from the same family often have identical or very similar binding specificities, and thus, as a consequence, many different PWMs may predict binding sites to multiple factors very well. Moreover, some motifs from public databases may represent the binding specificity of multimeric complexes involving other factors, and the identity of these other factors may be unknown, or not explicitly indicated by the authors of the PWM resource. For these and other reasons, we advocate an all-against-all (TF-versus-experiment) benchmarking approach and, for the first time, exemplify the merits of such an approach with results from a large-scale benchmarking study.

The potential benefits of all-against-all benchmarking are manifold, including:

- Identification of best performing PWMs for each factor, irrespective of PWM annotation in the source database
- Addressing the question of whether closely related members of the same TF structural family share the same binding specificity. This question can only be answered correctly if the motif comparison is coupled to the performance evaluation because otherwise, a difference in the motif accuracy (quality) could be misinterpreted as a difference in binding specificity.
- Coping with redundancy. Thousands of PWMs for human TFs are nowadays available from public databases. Eliminating suboptimal PWMs reduces this number to a few hundred while substantially increasing the average performance of the remaining matrices.
- Identification of all high-performance prediction targets of a given PWM. Being in possession of such a list would help biologists interpret motif matches returned by motif scanning programs. Today, most TF matrices are associated with a single TF, and naïve users of motif scanning software tend to believe that a reported match can only be bound by the TF appearing in the program output.
- Understanding in vivo TF-to-target site recruitment mechanisms. Several DNA binding TFs can also be recruited indirectly through protein-protein interactions. A classical example is STAT1, which upon gamma-interferon stimulation gets recruited to its native binding motifs (called GAS), whereas upon alpha-interferon stimulation gets recruited to another motif (called ISRE) via a multimeric complex that also includes STAT2 and IRF9 [23].
- Discovery of cooperative TF binding via motif co-localization. TFs may bind DNA as heterodimers recognizing a composite motif (e.g., TAL1-GATA1) or through contact-free modes of cooperation. For instance, pioneer factors may open chromatin to direct other TFs to cell-type-specific binding sites (e.g., FOXA1 guides ESR1).
- Gaining knowledge about tissue-specific motif activity. We observed that different unrelated motifs predict ChIP-seq peaks for a particular TF in different cell types. This may indicate different molecular mechanisms recruiting the same TF to different target sites already bound to DNA by another factor. Differential motif activity in this sense may help to predict TF binding sites in a tissue-specific manner using heterologous motifs.

## Results

Based on published work and our own experience, we have defined three benchmarking protocols for ChIP-seq, HT-SELEX, and PBM data. The three protocols are available in containerized form as docker images. The protocols for ChIP-seq and HT-SELEX data are also publicly accessible via a web interface. PBM data is publicly available via UniPROBE database [12].

### Protocol for ChIP-seq peak lists

The raw data from a ChIP-seq experiment are sequence reads. The first step in the analysis of the data is the mapping of reads to the genome. The next step is peak calling resulting in a peak list containing the coordinates of bound genomic segments at a resolution of about 200 bp. Our protocol starts with peaks and further requires binding strength-related quantitative scores assigned to peaks that can be used for ranking. As it will become clear later, the protocol requires a peak to be represented by a single position. To comply with this requirement, we take either the mid-point of the peak regions or, if available, the so-called summit position from the source peak files. We use only the *N* top-scoring peaks for benchmarking, extract the surrounding genomic sequences (+/− *w* bp), and score these sequences with the PWM under investigation, using the sum occupancy score as defined in [20]. Next, we score a set of negative control sequences of the same length, taken from genomic regions located at a fixed distance *d* upstream or downstream from the positive sequences. An area under the curve for the receiver operating characteristic (AUC ROC) value is then computed from the binding scores of the two sets, supposed to reflect the PWM’s capacity to discriminate between in vivo binding and non-binding sites.

### Protocol for HT-SELEX data

This protocol is applicable to all flavors of SELEX, which enrich a random pool of DNA oligonucleotides for sequences with high affinity to a particular DNA binding protein of interest. The data from such an experiment consist of a library of DNA sequences of a constant length (typically 14–40 bp). Current high-throughput SELEX technologies produce millions of sequences per experiment. As with the ChIP-seq peak-based evaluation method, we need a negative sequence set. It can be obtained by shuffling the positive sequences. Alternatively, sequences from the input library used in the protein-DNA binding reaction may be available for this purpose. From this point on, we proceed in a similar way as with the ChIP-seq peak lists. We first compute sum occupancy scores for all sequences in both libraries. However, because SELEX libraries are often only weakly enriched with true binding sequences (Additional file 1: Fig.S1, see also Fig. 1 in [24]), we take only a top percentile of the positive and negative scores (e.g., the top 10%) for ROC AUC value computation. Importantly, before PWM scoring, we extend the random insert sequences obtained from the sequence repository with the primer and barcode sequences that were present (and thus accessible to proteins) during the SELEX experiments.

### Protocol for PBM data

To assess the performance of PWMs on in vitro PBM data from the UniPROBE database [12], Pearson correlation values between normalized log probe intensities and log sum occupancy scores (see the “Methods” section) were computed per pair of PWM and PBM experiment.

### Overview of benchmarking study

The above-described protocols were used to benchmark 4972 PWMs characterizing binding specificities of human TFs from JASPAR [7], HOCOMOCO [11], and CIS-BP [13] against 2017 ChIP-seq peak lists from ReMap [25], 547 HT-SELEX experiments from [26] and [27], and 597 PBMs from UniPROBE [12]. ReMap ChIP-Seq peak lists included only human TFs data, whereas in vitro data from HT-SELEX contained samples from both human and mouse. The PBM data sets downloaded from UniPROBE were filtered for (i) belonging to human and mouse TFs but (ii) excluding non-wildtype TFs or technical variations of the experiment, see Additional file 7 for a complete list of retained experiments. Mouse data were mapped to the orthologous human TFs for the identification of the best performing motif matrix for a given TF.

Only peak lists containing at least 5000 peaks were considered. The benchmarking with SELEX data was done twice, with top-score cut-offs of 10% and 50%, in order to account for different degrees in true binding site enrichment of individual SELEX libraries. In total, this study produced a database of over 18 million performance values for experiment-PWM combinations, which we offer as a public resource. All results presented further below are based on this resource.

### Benchmarking reveals similar binding specificity across many but not all TF structural families

The ability of a motif to recognize peaks from a ChIP-seq experiment targeted at another TF of the same structural TF family is a long-disputed issue [28]. To quantitatively assess this family-related cross-binding, we grouped the PWMs with regard to the structural class of the respective TF’s DNA binding domain as recorded in the CIS-BP [13] and TFClass databases [29]. CIS-BP provides a more compact view of the TF families, while TFClass allows separating particular subfamilies, which are of interest in huge and diverse families such as zinc finger TFs.

**Fig. 2:**
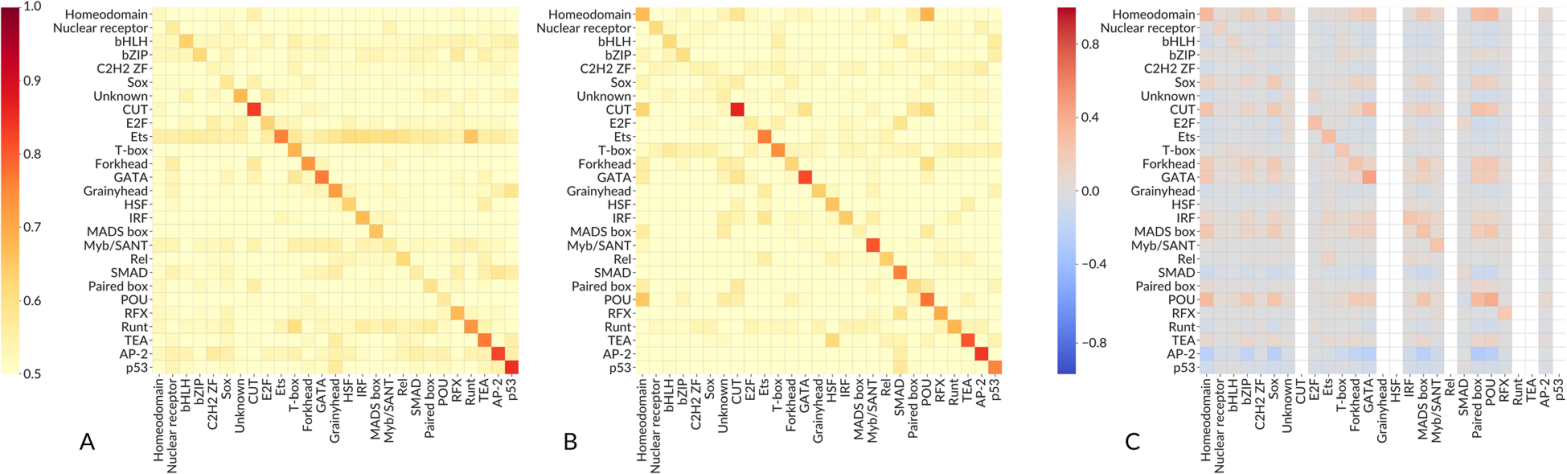
The average performance (**а**, **b** AUC ROC; **c** Pearson correlation coefficient) achieved by PWMs (rows) on particular data sets (columns). The average is taken over all binding motifs for TFs from the same family of DNA recognition domains according to the CIS-BP TF family classification and for all experiments for all TFs from the family of their DNA recognition domains. The PWMs were benchmarked on ChIP-seq (**a**), HT-SELEX 10% (**b**), and PBM (**c**) data. Only families with no less than 2 PWMs, 2 ChIP-seq, and 2 SELEX data sets are shown

ReMap ChIP-Seq peak lists included only human TFs data, and HT-SELEX and PBM included human and mouse data to increase coverage of TF families. In all cases, there is a clear signal at the diagonal showing that PWMs present in the databases indeed recognize TFBS of the corresponding TFs or at least of the TFs from the same CIS-BP family. Thus, on the global scale, the benchmark results agree with expectations based on DNA binding domain classification. Interestingly, there are families, e.g., “C2H2 zinc finger factors,” the TFs of which show almost negligible average family-wide AUC ROC on the corresponding data sets, both in vivo and in vitro, which agrees with their known diversity of DNA binding specificities. The results are consistent with the use of the 50% threshold in HT-SELEX benchmarks (Additional file 1: Figure S2A). The PBM-based heatmap (Fig. 2c) is sparse due to lower representation of TF families in the available experiments.

### Identifying the best performing matrix for each experiment and TF

**Fig. 3:**
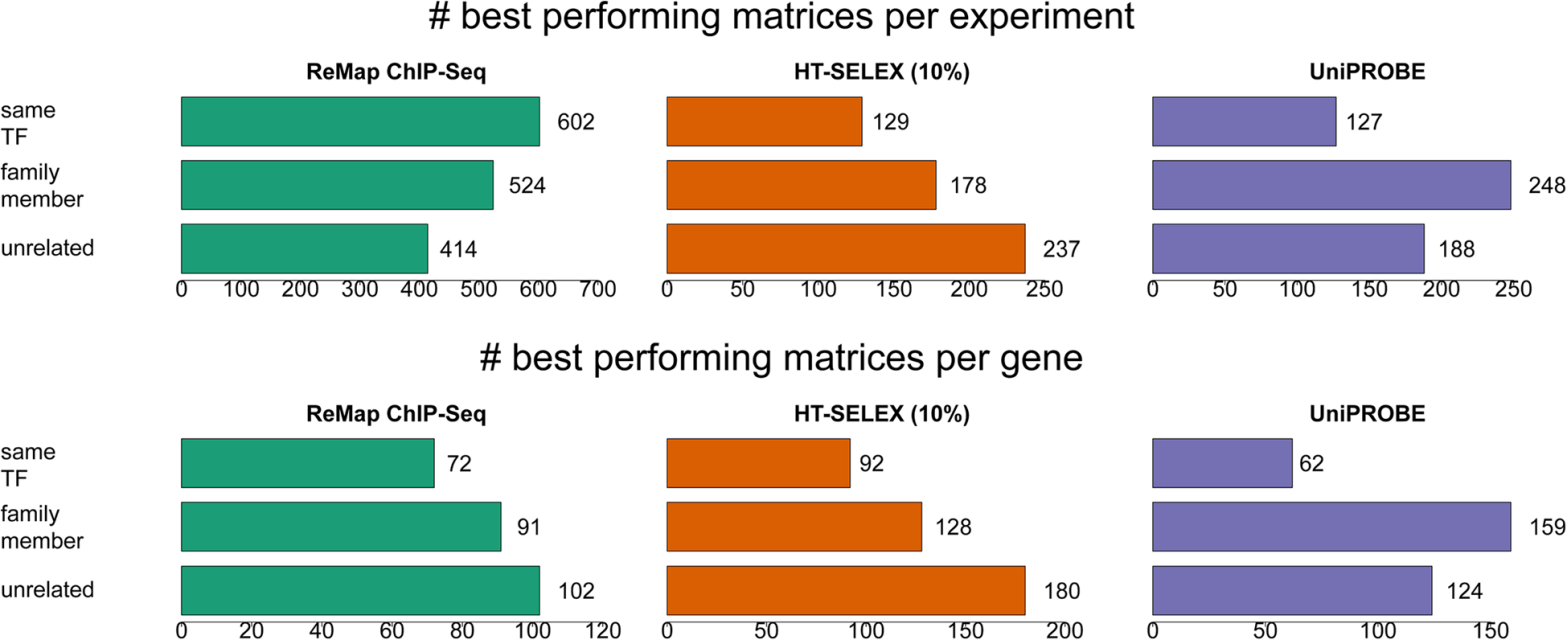
Statistics about best performing matrices. Four thousand nine-hundred seventy-two matrices from JASPAR, HOCOMOCO, and CIS-BP were benchmarked on ChIP-seq, HT-SELEX, and PBM data. The “best” matrix for a TF was chosen based on its aggregate rank score over all experiments attributed to this TF (see the “Methods” section)

It is well known that many TFs recognize similar binding sites due to the similarity of their DNA binding domains. Yet, direct motif comparison tells little whether two TFs of the same family recognize the same motif because motif differences can relate to experimental errors or other confounding factors such as base composition inhomogeneity of the genomic context of binding sites. To test this, we identified for each experiment the best performing matrix among all available PWMs. We also extracted the globally best performing matrix for each TF, defined as the matrix with the highest aggregate rank score (see the "Methods" section) over all experiments for the same factor (different types of experiments were analyzed separately). We further distinguished between cases where the best performing matrix was for a TF from the same or a different family according to TFСlass [29]. The statistics of the best performing PWMs classified according to their place in the TFClass hierarchy is presented in Fig. 3.

We observed that for most of the experiments, the best performing matrix was not attributed to the same factor, but in fact very often to a member of the same family. Quite surprising is the relatively high number of best performers coming from different TF families both for ChIP-seq and HT-SELEX experiments. However, a closer look at the results indicates that these matrices most often either are attributed to a TF family from the same structural class or show low performance on an absolute scale. In summary, the high-quality matrices tend to perform well for several TFs of the same family, sometimes even for other families of the same class.

**Fig. 4:**
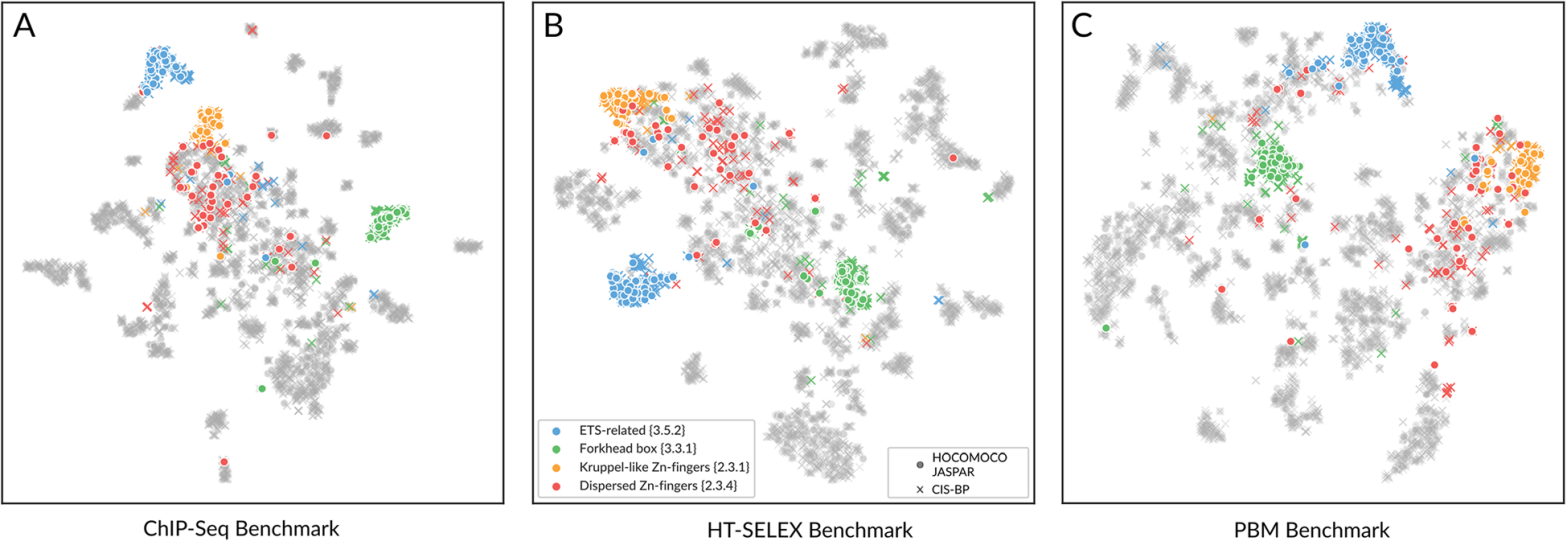
PWMs grouping according to the similarity of their performance measure values across data sets. PWMs recognizing bound regions in similar selections of experiments are grouped reasonably well according to their TFClass families. Dimensionality reduction with t-SNE is applied to motifs’ performance at ChIP-seq (**a**), HT-SELEX 10% (**b**), and PBM data (**c**). For illustration, several TF families are highlighted with color. Each point corresponds to a PWM. Source coordinates are AUC ROC values (**a**, **b**) or Pearson correlation coefficients (**c**) calculated for different data sets. ‘o’ HOCOMOCO and JASPAR PWMs, ‘x’ CIS-BP PWMs

Different PWMs for TFs from the same structural families tend to perform similarly across experiments. To explore this trend in detail for selected TF families, we applied t-SNE [30] to the complete matrix of AUC ROC values and distributed motifs on a 2D plane according to their tendency to recognize the bound regions in different data sets. The expectation was that motifs performing similarly on the same ChIP-seq experiments will be located close to each other. Indeed, the motif distribution at the t-SNE projections in general agrees with the structural classification of TFs. In Fig. 4 (see also Additional files 2, 3, 4, and 5 for interactive plots in which any family can be highlighted), each point corresponds to a PWM, and the PWMs for TFs from the selected illustrative families are colored with the same color. One can see that most of ETS and Forkhead motifs group together. On the other hand, motifs of the “factors with multiple dispersed zinc fingers {2.3.4}” are dispersed on the t-SNE plane, which agrees with low average family-wise AUC ROC values seen in Fig. 2. In contrast, motifs for three-zinc finger Krüppel-related factors {2.3.1} binding CCCCG-boxes are nicely clustered together.

The clustering patterns for motifs for TFs from the same structural families do not depend on the type of experimental data (e.g., ChIP-seq or HT-SELEX) or on the TF motif collection (e.g., JASPAR or HOCOMOCO). Also, we did not observe any dependence on the TF classification resource used; results for CIS-BP families agreed well with those for TFClass, see Additional files 2, 3, 4, and 5.

For most of the TFs, the best performing matrix comes from the same structural family. Yet, there exist some exceptions, which can be readily visualized by plotting motif-experiment best-performance pairs at alluvial plots.

**Fig. 5:**
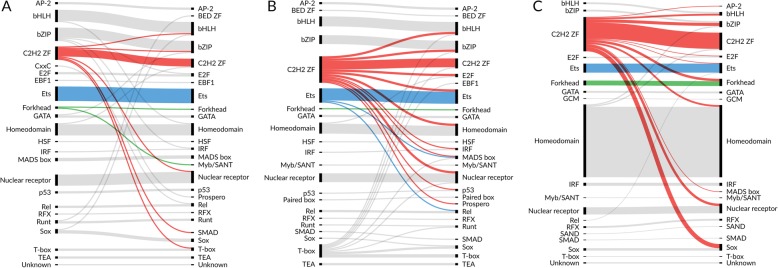
Alluvial plots illustrating the performance of PWMs from particular CIS-BP TF families in ChIP-seq (**a**), SELEX 10% (**b**), and PBM (**c**) benchmarks. For each TF, PWMs with the highest average AUC ROC (**a**, **b**) or Pearson correlation coefficient (**c**) across the data sets for this TF are used to construct the links. PWMs grouped by CIS-BP TF family are shown on the left; TFs are shown on the right. The link width corresponds to the number of TFs. Only TFs with at least one ChIP-seq data set and one HT-SELEX data set are included. For illustration, selected motif families are highlighted with color

Figure 5 shows many cases of individual TFs with the best average performance obtained for TFs for other structural classes for ChIP-seq (A), HT-SELEX 10% (B), and PBM (C) benchmarks (for HT-SELEX 50%, see Additional file 1: Fig.S3). This is particularly true for C2H2 zinc fingers, many PWMs of which often recognize TF binding peaks for different TF families. Interestingly, in most other cases, ChIP-seq peaks or HT-SELEX oligos were recognized by TFs belonging to the same TF family. Most of the cross-family recognition for C2H2 zinc fingers are likely to be explained by random errors in motif building or, in the case of ChIP-seq, by TF-TF interactions, where the target TF is bound to DNA through another TF acting as a mediator. Another cross-family example is given by a low-complexity (probably incorrect) polyG TBX15 motif of T-box factors that achieves the best performance for several other TFs in the HT-SELEX benchmark only. This might be an artifact arising from the nucleotide composition of the HT-SELEX input control libraries that were used as a negative control set.

**Fig. 6:**
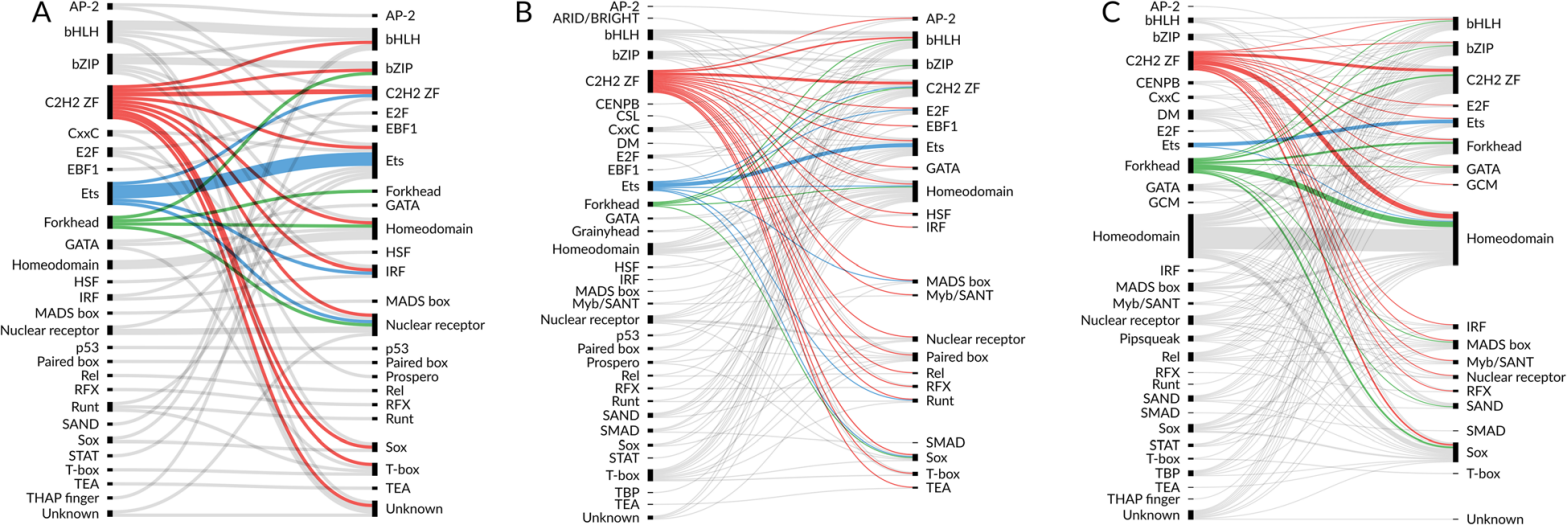
Alluvial plots illustrating the performance of PWMs from particular CIS-BP TF families in ChIP-seq (**a**), SELEX 10% (**b**), and PBM (**c**) benchmarks. For each TF, PWMs displaying the average AUC ROC of no less than 0.75 (**a**, **b**) or Pearson correlation coefficient of no less than 0.3 (**c**) across the data sets for this TF are selected for link construction. PWMs grouped by CIS-BP TF family are shown on the left; TFs are shown on the right. The link width is proportional to the square root of the number of appropriate PWM-TF pairs. Only TFs with at least one ChIP-seq data set and one HT-SELEX data set are included. For illustration, selected motif families are highlighted with color

The cross-family recognition patterns become much noisier when all the cases reaching an average 0.75 AUC ROC or average 0.3 Pearson correlation are taken into account rather than the best average performers (Fig. 6 and Additional file 1: Fig.S3B). In this setting, cross-family prediction rates increase significantly, suggesting that partial overlaps in the consensus sequences allow reaching significantly higher-than-random recognition quality both when using in vitro and in vivo data and thus highlighting the need for comparative assessment of multiple motifs.

### Making a small, non-redundant, quality-filtered PWM library

Recently introduced high-throughput TF binding assays have generated a wealth of data, from which DNA binding specificity models can be built. As a result, the number of TF binding matrices has exploded, and fighting redundancy has become an issue of great practical importance. If the binding specificity of several TFs can be explained by the same motif, it will be possible to significantly reduce the number of binding specificity models while still covering the same number of TFs.

**Fig. 7:**
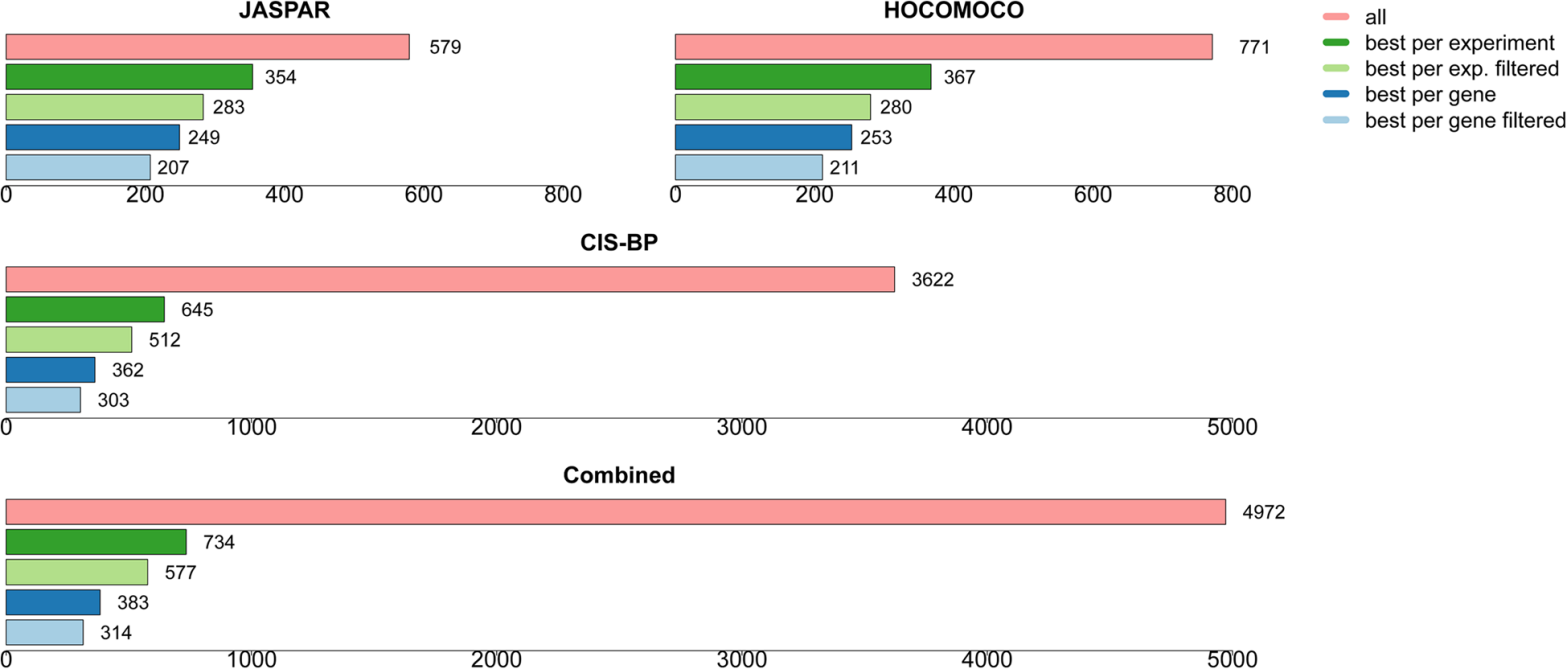
Statistics on the best performing TF motif matrices. “Best performance per gene” means globally best performance over all corresponding ChIP-seq, HT-SELEX (top 10%), and PBM experiments in terms of aggregate rank scores (see the “Methods” section) over all corresponding experiments. The qualifier “filtered” relates to the numbers obtained when we only considered experiments for which at least one matrix achieved a ROC AUC value > 0.75 (ChIP-seq, HT-SELEX) or a Pearson correlation coefficient > 0.35 (PBM). The first three bar plots show the numbers for individual motif collections analyzed separately, whereas the last plot at the bottom shows the numbers obtained when all three collections were considered simultaneously

One way of making a non-redundant motif library for TFs is by choosing the best performing matrices for each TF (Fig. 7). This approach reduces the number of motifs more than a tenfold from 4972 to 414 (339 if only high-quality matrices with AUC ROC > 0.75 or Pearson correlation coefficient > 0.35 are considered) while increasing the average predictive performance for each TF at the same time.

As SELEX data are qualitative (binding versus non-binding) and PBM data quantitative (relative binding affinities of different sequences), we were wondering whether matrices performing well in a classification-based test were able to predict normalized signal intensities from a PBM experiment. Examining several cases, where a TF has been assayed by at least two different techniques, and multiple matrices were available for the same TF, led us to conclude that this is indeed the case. An example is presented in (Additional file 1: Fig.S4) where the ROC AUC values of 12 different PWMs for FOXJ3 obtained on HT-SELEX data were compared to corresponding correlation coefficients obtained on PBM data. The two number series are highly concordant with a correlation coefficient of 0.78.

The alternative approach to reduce redundancy is exemplified by the widely used methods for identifying representative motifs by clustering. Motif clustering is a usual procedure in many applications. In motif activity response analysis [31], TFs with similar binding specificities bring about mathematical difficulties, so it is necessary to reduce the set of considered motifs to a subset of dissimilar motifs. As a result of motif clustering, a representative motif is often selected or constructed for a set of similar known motifs. Binding specificities of untested TFs can be predicted by the similarity of DNA binding domains [32].

**Fig. 8:**
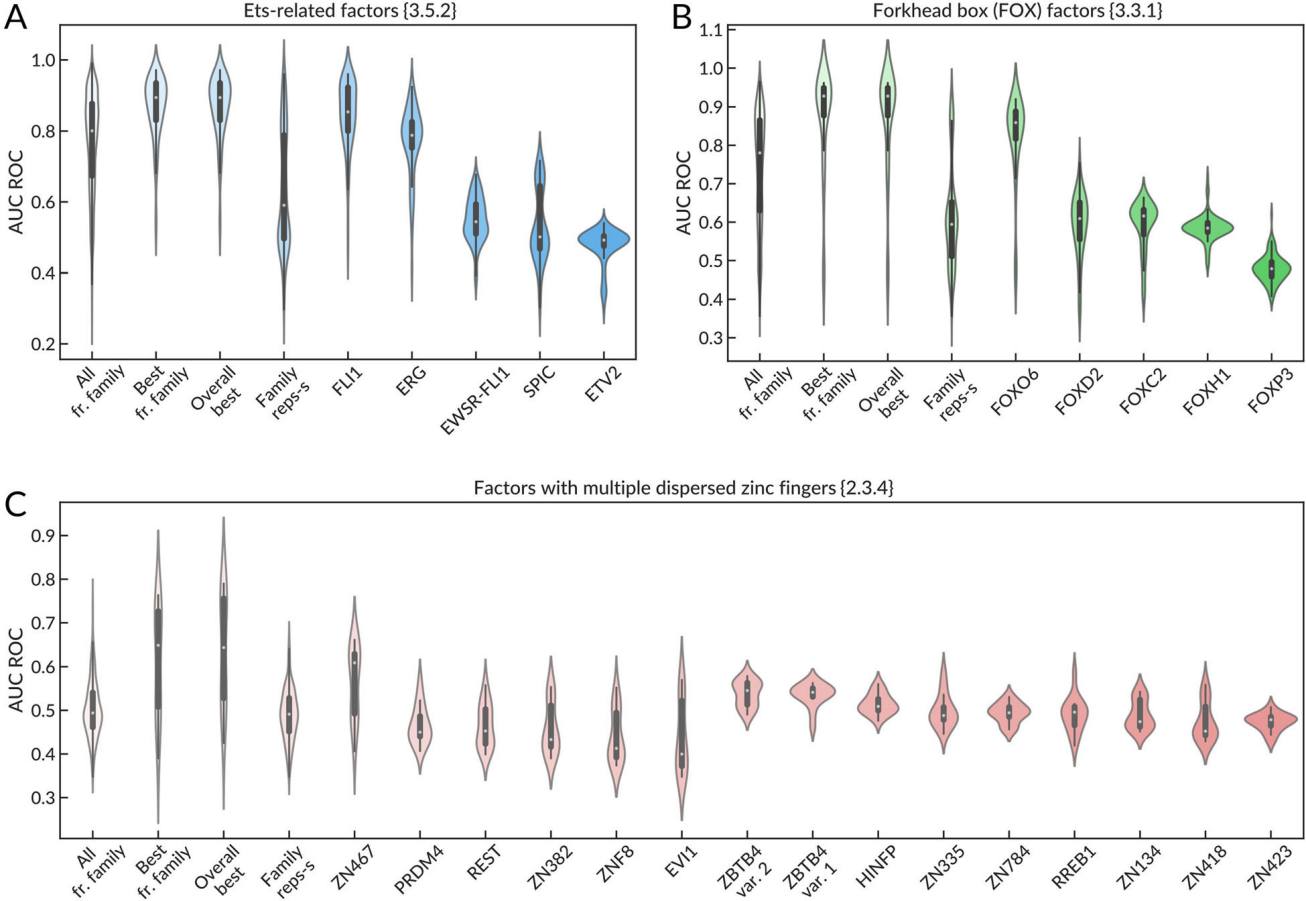
Violin plots of AUC ROC values obtained on ChIP-seq data sets for TFs of a particular TFClass family by PWMs belonging to TFs of the same family and representative motifs of the family selected using the PWM clustering-by-similarity. **a** Ets-related factors, **b** Forkhead box (FOX) factors, and **c** factors with multiple dispersed zinc fingers. All AUC ROC values are obtained using data sets of TFs from the selected family. The first 4 violins of each plot show AUC ROC values of (1) all PWMs of TFs from the selected family, (2) the best PWM (with the highest average AUC ROC) from the family, (3) the best PWM from all tested, and (4) family representatives obtained by the motif similarity clustering. The next violins of each plot show AUC ROC values achieved by particular representative PWMs belonging to the family and selected from the motif clustering by similarity

For motif clustering, we used a benchmark-blind approach similar to that used in [33], with PWMs from HOCOMOCO and JASPAR databases. The systematic comparison of performance metrics allowed us to assess the effectiveness of the two different strategies, selecting the best representative motif for a motif similarity cluster or selecting the best performing motif within a TF structural family. In Fig. 8, we show the applicability of representative motifs for the problem of binding site recognition in a selected TFClass structural family on three examples: Ets-related factors {3.5.2}, Forkhead box (FOX) factors {3.3.1}, and factors with multiple dispersed zinc fingers {2.3.4}.

The performance of the family-specific matrices selected by different approaches is visualized as violin plots. It turned out that “the best from the structural family” or “the best globally” matrices generally outperform “the representatives from family” matrices derived by clustering. Indeed, Fig. 8 displays that while some representative motifs (e.g., in FOX or ETS families) displayed good TFBS recognition, close to the best available PWMs, the other had mediocre or even “random-guess” (AUC ROC of 0.5) performance. There are many more representative PWMs for dispersed zinc finger proteins, which can be explained from their diversity (see Figs. 2 and 4). Not surprisingly, in this case, none of the representative PWMs is able to properly recognize TFBS across the entire family.

The high level of within-family cross-recognition is exemplified in Table 1 where a single PWM (MA0028.2 from JASPAR) is the best predictor for multiple TFs from the ETS family. In JASPAR, this DNA motif is assigned to ELK1. All TFs, for which this matrix reaches AUC ROC > 0.75 in at least one ChIP-seq or HT-SELEX experiment, are shown. An exclamation sign indicates that this matrix is the overall best performer for the corresponding factor (see table legend). Regarding ChIP-seq in vivo experiments, this matrix is the best performer for as many as 10 different TFs, only 5 of which are ETS family members. Surprisingly, it is also the best performing matrix for 5 unrelated TFs, including BRCA1. Note further that the list of top-ranked TFs includes three histone-modifying proteins (JMJDC1, SUZ12, KDM5A) and two cycline-dependent kinases (CDK9, CDK7). We suspect that these unrelated factors could be recruited to their target binding sites through protein-protein interactions with a DNA-bound ETS factor.

**Table 1:**
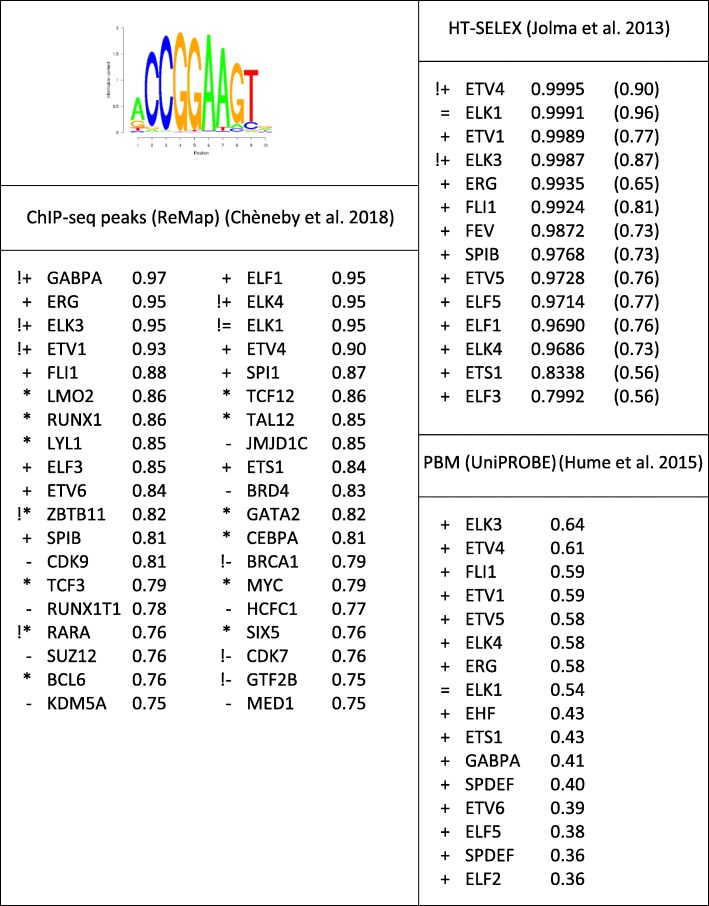
Performance report for MA0028.2, gene ELK1, and family 3.5.2 Ets related

The numbers indicate the highest performance value achieved by the JASPAR MA0028.2 matrix for any experiment targeted at the corresponding TF (identified by gene symbol). Only genes with AUC ROC > 0.75 or Pearson correlation coefficient > 0.35 are listed. For HT-SELEX experiments, the numbers in parenthesis represent ROC AUC values obtained with a top-score cut-off of 50% instead of 10% (see the “Methods” section)

Meaning of symbols: !, MA0028.2 is overall best performing matrix for this DNA binding TF; =, designated target of MA0028.2; +, from the same TFclass family (3.5.2 Ets-related factors); *, from another TFclass family; −, missing in TFclass (often a chromatin protein without DNA binding domain)

The corresponding list for HT-SELEX experiments looks very different. There, ELK1 appears on the second place of the list, preceded and followed exclusively by other members of the ETS family. JASPAR MA0028.2 is the best performing matrix for ETV4 and ELK4, but surprisingly not for ELK1, where it is outperformed by JASPAR MA0765.1 assigned to ETV5 (AUC ROC 0.9998). The extremely high-performance values for most HT-SELEX data sets suggest that the majority of ETS members have indistinguishable DNA binding specificity. Also with PBM data, this matrix shows good performance only for ETS family members.

### Comprehensive benchmarking helps clarify the molecular meaning of binding motifs

PWMs from public resources are typically assigned to a single TF (single polypeptide chain) identified by a gene symbol. This TF usually corresponds to the target of the antibody used in the experiment. However, with regard to motifs derived from ChIP-seq data, the role of the motif in the recruitment of the TF to target sites remains essentially unknown. It is possible that the motif represents the binding specificity of another TF, which tethers the TF targeted by the experiment to the ChIP-seq peak regions through protein-protein interactions. Several such cases are well documented by experiments. Two cases where performance reports help clarify these respective roles of dissimilar motifs in TF recruiting are presented in Tables 2 and 3.

**Table 2:**
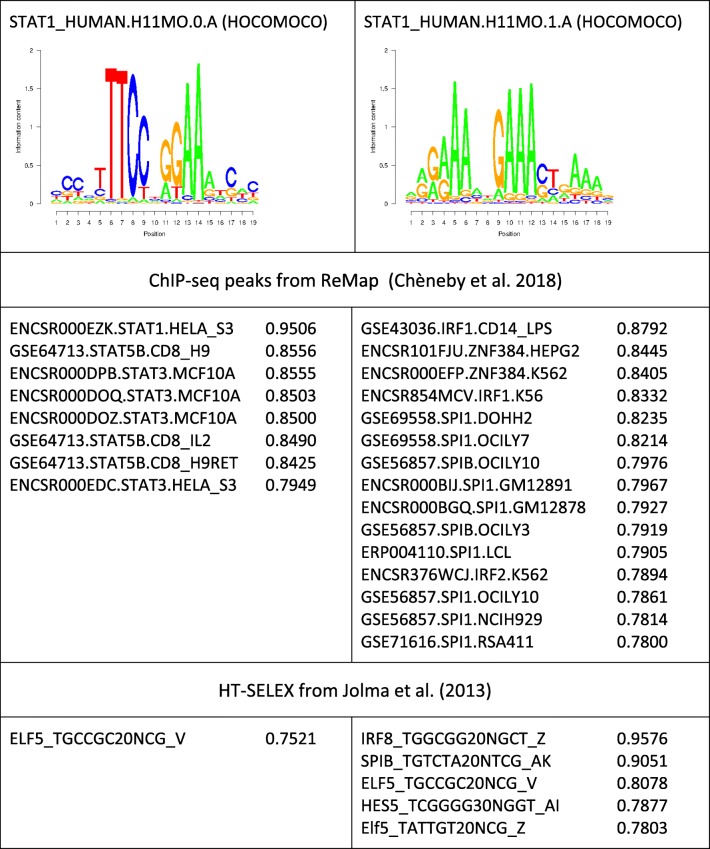
Performance summary of two STAT1 matrices

Only experiments with AUC ROC > 0.75 are shown. No PBM experiments reached a Pearson correlation coefficient > 0.35

**Table 3:**
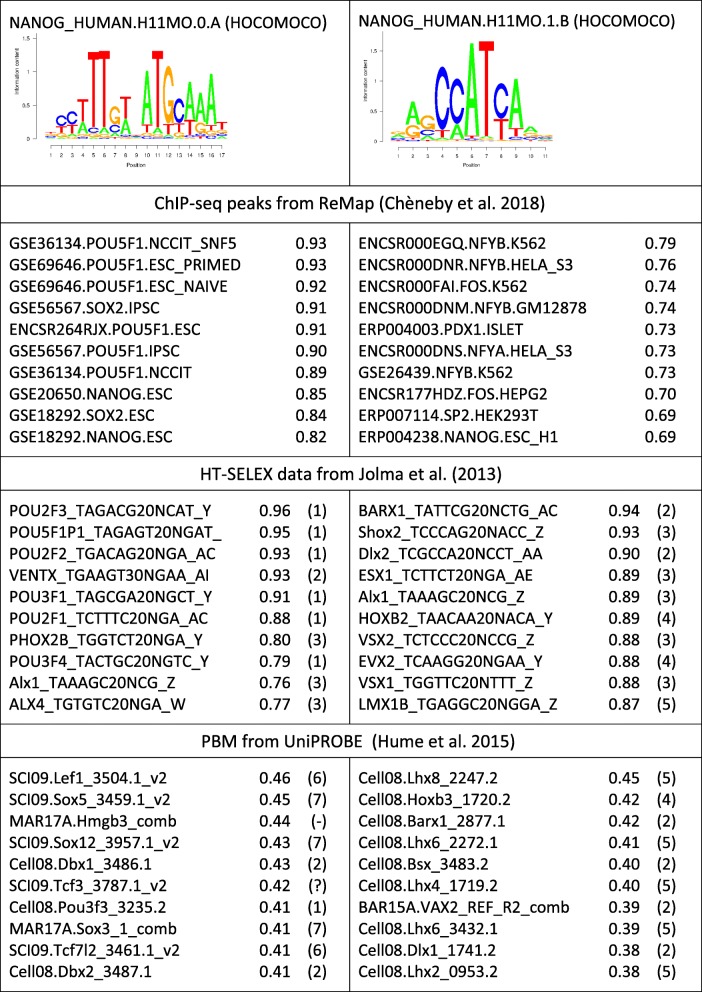
Performance summary of two NANOG matrices

Only the 10 highest scoring experiments are shown. For HT-SELEX and PBM experiments, the TF’s family affiliation as defined in TFClass is shown in parenthesis: (1) POU domain, (2) NK-related (including NANOG), (3) paired-end, (4) HOX-related, (5) HD-LIM, (6) TCF-7-related, (7) SOX-related, (?) possibly outdated TF name (may in fact be Tcf7l1), and (−) unclassified

Table 2 shows a comparison between two alternative matrices for STAT1 from HOCOMOCO. We first note that the motif logos are quite different, the one on the left side being near palindromic, and the one on the right side being composed of tandem repeats. Regarding ChIP-seq experiments, we note that the former reaches high-performance values (AUC ROC > 0.75) only for members of the STAT family, whereas the latter shows good performance for the unrelated proteins IRF1/2, ZNF384, SPIB, and SPI1. Regarding HT-SELEX data, the tandem-repeat motif performs best on an experiment targeted at IRF8. The HT-SELEX results for the palindromic motif are not congruent with the ChIP-seq results. This is however not surprising as the HT-SELEX collection does not include any data set for a STAT family member.

Overall, the results are consistent with models derived from experimental studies: (i) Upon alpha-interferon induction, STAT1 forms a complex with STAT2 and IRF9, which binds to a target DNA motif called ISRE [23]; (ii) IRF8 was found to form a complex with SPIB, which then binds to an Ets/IRF composite element [34]. Taking together, these previous findings and our results suggest that the STAT1 matrix, whose logo is displayed on the right side, likely represents the intrinsic DNA specificity of 2 or 3 IRF family proteins arranged in tandem fashion.

Table 3 shows benchmarking results for two alternative NANOG matrices. This is another well-known example of alternative TF-to-DNA recruitment pathways for the same TF. Nanog reportedly can bind indirectly to DNA by associating with a SOX2-POU5F1 (Oct4) dimer bound to a composite motif; alternatively, Nanog can also directly bind to DNA via its own homeo domain [35, 36]. The results shown in Table 3 suggest that the matrix on the left side represents the indirect binding mode and the matrix on the right side the direct one. The composite motif matrix shows good performance for all three TFs on corresponding ChIP-seq data. It also performs well on HT-SELEX data for POU5F1 and on PBM data for SOX-related proteins. The matrix on the right side shows good performance on ChIP-seq data for general promoter-binding factors (NFY, SP1/2) and (with somewhat lower AUC ROC values) for Nanog itself. Unfortunately, the HT-SELEX and PBM collections do not include data for Nanog. However, the fact that the top-ranked HT-SELEX experiment, and 4 out of 10 top-ranked PBM experiments are assigned to a TF from the same homeodomain subfamily as Nanog (NK-related), speaks in favor of the hypothesis that this matrix represents the intrinsic DNA binding specificity of Nanog. In contrast, the majority of the top-ranked HT-SELEX experiments for the other motif relate to POU domain factors.

Recently, a modified HT-SELEX protocol has been published [37], enabling the characterization of TF heterodimers. We were wondering whether the hypothesized indirect binding target of Nanog mediated by a SOX2-POU5F1 dimer was supported by new data generated with this technology. This is indeed the case. The authors of the above-cited paper were able to extract a motif for a closely related complex, a SOX2-POU2F1 TF pair (Additional file 1: Fig.S5), which closely resembles the sequence logo shown on the left side of Table 3.

## Discussion

We have generated the first data set of all-against-all performance measures for three human PWM libraries and three reference experiment collections, one in vivo and two in vitro. The results presented here are only the first deliverables of an ongoing benchmarking initiative, which we plan to extend to more PWM resources and non-human model organisms in the near future. Nevertheless, clear trends have already become apparent, which have important implications for the research field.

As suspected (but never formally proven by systematic testing), many human TFs have indistinguishable binding specificity, at least at the resolution of current experimental techniques. In fact, in a majority of cases, the best matrix for a given TF was not derived from experiments targeted at the same TF but very often from another member of the same family. Comprehensive benchmarking allows the identification of good quality matrices, which often perform indistinguishably well for several TFs.

Our study also highlights exceptions to the rule that members of the same TF family have similar binding specificity. For instance, some promiscuity is observed between different families of the homeodomain structural class. Perhaps, our benchmarking results could be used to improve the TF classifications. This, of course, raises the controversial question of whether TF classification should be based on DNA binding domain structure, phylogeny, or DNA binding function. In any case, the comparison between sequence- and performance-based classifications could help define or refine rules and algorithms to predict DNA binding specificity from protein sequence.

It is quite likely that the practical set of motifs for many biological applications would be much smaller than the number already contained in the most popular collections. Interestingly, we failed to find any reasonable property, associated with best performing motifs; they displayed rather uniform distribution in terms of motif length or Information Content per position (see kernel density plots in Additional file 1: Fig.S6), and only PBM benchmark results displayed general GC content bias.

The performance measures relating to ChIP-seq data help elucidate molecular mechanisms of gene regulation. We were generally surprised how many matrices performed well on experiments targeted at unrelated proteins, either TFs from other structural classes or non-DNA binding chromatin proteins. Many cases of such cross-performances may reflect in vivo protein-protein interactions. Some TFs may jointly bind to DNA as heteromeric complexes. In other cases, a TF, which has the capacity to directly bind DNA, may be recruited to some target sites via binding to another TF, without contacting the DNA. Comparison with in vitro benchmarking results can in such cases help discriminate between alternative hypotheses.

A surprising observation regarding results presented in Figs. 5, 6, and 8 is that the benchmarking using in vitro and in vivo data is very consistent at the global scale of TF families, suggesting, despite principal differences of in vivo and in vitro binding, there is a major quantitative agreement between different data types.

The aim of our initiative was to evaluate the quality of TF specificity models in terms of binding site recognition and affinity prediction. However, during the course of this study, we realized that the performance measures obtained by our benchmarking protocols were equally indicative of the quality of the experimental data sets used. Some data sets consistently produced low AUC ROC matrices, even with the best performing PWM for the corresponding TF (for an example, see Additional file 1: Fig.S4). Our public benchmarking results thus can be used for retrospective quality assessment of the experimental test data used.

We have designed, implemented, and productively used three different benchmarking protocols in this study. In our assessment, these protocols produce biologically meaningful results, which are remarkably consistent across experiments. An important novelty of our approach is that we include primer and barcode sequences in the evaluation of HT-SELEX data, in order to account for overlapping binding sites. For instance, the barcode of the HT-SELEX library named “ELK1_TCGGAA20NAGT” provides the 3′ terminal ATG of the ELK1 motif (CCGGAATG) and as a consequence contains many inserts ending with TCGGA. These binding sites are missed if only inserts are scanned. In almost all cases, including primers and barcodes improved the AUC ROC values obtained with the matrices for the same or a related TF, especially for long PWMs. To facilitate the usage of the same protocols by others and to ensure reproducibility, we made these protocols available as docker images.

Despite the encouraging results obtained so far, we believe there is room for additional PWM benchmarking protocols. We have already in place a method for evaluating TF binding motifs directly with ChIP-seq read mapping data, using BAM or BED files as input. The performance measure returned is a central enrichment score for the read-density around top-scoring motif matches from a whole genome scan. This protocol has the principle advantage that it starts closer to the raw data, bypassing the peak finding step, which adds an additional source of variation influencing the performance score. As an alternative to our current peak-list-based method, we may in the future consider the motif-enrichment-based approach proposed in [21], which we find conceptually appealing.

There is also room for improvement for the existing protocols. For the ChIP-seq peak-list-based method, there may be better choices for the negative control set. For instance, one could try to match positive and negative genomic regions in terms of properties such as chromatin accessibility or nucleosome occupancy (if data are available for the corresponding cell type). For HT-SELEX data, an automatic and objective way of determining the optimal top score threshold would be desirable, to make results from libraries with varying levels of binding site enrichment more comparable.

A potential criticism of our study design relates to potential circularity and over-fitting effects arising when a PWM is benchmarked on the data, from which it was originally derived. Unfortunately, the matrices from public databases are not necessarily linked to source data. Even CIS-BP matrices annotated as being derived from HT-SELEX or PBM cannot easily be traced to individual experiments when multiple experiments have been carried out for the same TF. Thus, we cannot know which performance values may be inflated by over-fitting effects. However, we think this problem is not so critical for this study. For most TFs, there are multiple test sets in our collection. Users of our benchmarking results can thus assess the quality of a given matrix based on the robustness of its performance across multiple data sets. Furthermore, the majority of best performing matrices for a given experiment are assigned to a different TF in the source databases. In all these cases, the best performing matrices were obviously derived from a different experiment, indicating that over-fitting is not a major source of bias in this study.

The benchmarking of the representative motifs obtained from benchmarking-blind motif clustering underlines the risks and challenges of reducing the PWM library complexity by straightforward clustering PWMs by similarity and selecting the representative members as cluster centers. We believe that, at the current volume of data available, such procedures are becoming increasingly obsolete and are likely to be substituted with direct benchmarking of alternative motifs on several experimental data sets.

## Conclusions

We have carried out a comprehensive all-against-all PWM-to-experiment benchmarking study, resulting in the computation of more than 18 million performance measure values. We make these numbers available as a public resource, in a format that can easily be imported into statistical analysis software, such as the R environment, for further exploratory analysis. We have shown by selected examples and statistical plots that our resource is useful for selecting an optimal matrix for a particular purpose, as well as interpreting motif matches reported by a DNA sequence analysis program. The PWM performance spectrum for a given ChIP-seq experiment sheds light on the various TF-to-target site recruitment processes active in a particular cell type under particular conditions. We consider extending this resource in the future but are already confident that it will be of great value in its present state.

## Methods

### Origin and preprocessing of PWM libraries

Base probability matrices from JASPAR Core Vertebrate (2018) and HOCOMOCO Human (v11 FULL) were downloaded in MEME database format from the MEME suite web server. CIS-BP matrices were directly downloaded from the original web site. The CIS-BP collection used in this work contains all matrices directly inferred from experiments with human TFs except those matrices imported from JASPAR and HOCOMOCO. Matrices were manually mapped to gene symbols and TF families from TFclass [29] and CIS-BP. The annotations of matrices (including basic features such as length and GC content) and respective TFs are provided in Additional file 6. The original matrices were slightly modified by adding a correction term of 0.0001 to each matrix element, followed by renormalization of the position-specific probability distributions. This preprocessing step serves to prevent numerical exceptions due to logarithms of 0. Incidentally, we also found that it increases benchmark performance for almost all matrices.

### Benchmarking with ChIP-seq peak lists

A special version of ReMap peak lists [25], which included signal enrichment scores, was used in this work and can be downloaded from the MGA data repository [38]. Only peak lists with at least 5000 peaks were used. The following parameters were used for benchmarking: region width *w =* 250, number of top-ranked peaks: *N* = 2000, location of negative control sequence relative to peak centers *d =* + 500.

### Benchmarking with HT-SELEX data

Library sequences described in [26] and [27] were downloaded from the European Nucleotide Archive ENA [39] in FASTQ format. The source files contain the DNA sequences of the random inserts without barcodes or primers. FASTQ files were converted into FASTA format. Only sequences exclusively composed of A, C, G, and T and having the length indicated by the library name were retained.

Note that the sequences from the two studies were derived from the same series of experiments. Libraries representing different cycles from the same experiment were pooled. Duplicates were then eliminated from the pooled libraries (assuming that they were PCR copies from the same founder molecules). A random subset of one million sequences was extracted from libraries containing more than a million sequences, in order to reduce benchmarking computing time. As an alternative to input (zero-cycle) sequences, we also generated negative control sequences by mononucleotide shuffling of the inserts but leaving the primer and barcode sequences unchanged (see below).

We extended the random insert sequences provided in the source files with sequences that were physically present during the binding experiments. According to [26], the 5′ and 3′ flanking primer sequences were as follows:

5′ TCCATCACGAATGATACGGCGACCACCGAACACTCTTTCCCTACACGACGCTCTTCCGATC

3′ ATCGTATGCCGTCTTCTGCTTGCCGACTCCG

The barcodes vary from experiment to experiment. After flanking with barcode and primers on both sides, sequences were truncated to include only the 20 bp adjacent to the random insert on each flank. For instance, random insert sequences from experiment ELK3_TCGGGG20NGGT_AG (barcodes TCCGGGG and GGT, as indicated by the name) were extended in the following way:

ACGCTCTTCCGATCTCGGGGNNNNNNNNNNNNNNNNNNNNGGTATCGTATGCCGTCTTCT

As the source files containing the ReMap peak lists and SELEX libraries have informative and intuitively understandable names, they were thus used as experiment identifiers in this article. The TF gene symbols assigned to each experiment were also extracted from the corresponding filenames.

### Benchmarking with PBM data

Protein-binding microarray (PBM) data for human and mouse were downloaded from UniPROBE (Hume et al. [12]) as “Normalized Probe Data.” The files for each experiment contain one column with normalized intensities per probe and the actual probe sequence including a fixed linker sequence. To obtain correlation values for an input PWM (obtained from JASPAR, HOCOMOCO, or CIS-BP and processed as described previously) with respect to measured log intensities, the following procedure was adhered to: For each probe sequence, the first 41 base pairs were extracted, excluding part of the fixed linker sequence. Within this sequence, the PWM was applied in a sliding window approach to obtain per-position probabilities, which were aggregated into a log sum occupancy score, i.e., the log of the sum of these probabilities per probe sequence. Finally, the Pearson correlation coefficient between the log sum occupancy scores and log intensities was reported as correlation value for a pair of PWM and PBM experiment.

### Benchmarking protocol availability

The ChIP-seq and HT-SELEX-based benchmarking protocols are publicly available via a web interface at https://ccg.epfl.ch/pwmtools/. The ChIP-seq, HT-SELEX-based, and PBM-based protocols are available as docker images from https://github.com/autosome-ru/motif_benchmarks [40].

### Aggregate rank score and identification of the best performing matrix for a gene

All rows corresponding to a given gene were extracted from a complete table containing performance measures (ROC AUC or correlation coefficients) for combinations of experiments (rows) and TF motif matrices (columns). The numbers in a given row were first converted into ranks. The overall performance of a matrix for multiple experiments for the same gene was then computed as the geometric mean over the ranks. This score is referred to as the “aggregate rank score” elsewhere in the text.

### Motif clustering

For motif clustering and selection of representative motifs, we first constructed a distance matrix between all pairs of human TFs in HOCOMOCO and JASPAR, using MacroAPE [41] to calculate Jaccard distances between sets of words recognized by motifs. This distance matrix was used to make a hierarchical tree using UPGMA (unweighted pair group method with arithmetic mean). Motif aggregation halted when the distance between merging clusters reached 0.95 or more. As a result, we came up with 225 clusters. From each cluster, one representative motif was taken by minimizing its average distance to other motifs in the cluster.

### T-SNE analysis

The analysis was performed using the sklearn t-SNE implementation [42] with PCA initialization. Cosine similarity was used as a distance parameter. The perplexity parameter was set as 25 for ChIP-seq and HT-SELEX benchmarks and 55 for the PBM benchmark.

### Analysis of the association between the basic motif features and the performance values

Each point underlying the density plot corresponds to a single PWM of a particular TF. To normalize for different numbers of data sets per TF, the AUC ROC (for ChIP-seq and SELEX) and correlation (for PBM) values were calculated by averaging the corresponding values over all data sets for the TF corresponding to the PWM under consideration. The Python seaborn package was used for visualization.

The complete benchmarking results are available from https://github.com/autosome-ru/motif_benchmarking_data [43].

## Supplementary information

**Additional file 1.** Supplementary figures and text.

**Additional file 2.** Interactive t-SNE plots for ChIP-seq benchmarks.

**Additional file 3.** Interactive t-SNE plots for HT-SELEX (cut-off 10%).

**Additional file 4.** Interactive t-SNE plots for HT-SELEX (cut-off 50%).

**Additional file 5.** Interactive t-SNE plots for PBM benchmarks.

**Additional file 6.** Annotation of TFs and motifs.

**Additional file 7.** List of PBM experiments used.

**Additional file 8.** Review history.

## Data Availability

JASPAR matrices: http://jaspar.genereg.net HOCOMOCO matrices: http://hocomoco11.autosome.ru/ CIS-BP matrices: http://cisbp.ccbr.utoronto.ca/ ReMap peak lists: http://remap.cisreg.eu HT-SELEX data: https://www.ebi.ac.uk/ena/data/view/PRJEB3289 (+PRJEB14744) PBM data: http://thebrain.bwh.harvard.edu/uniprobe/ Docker implementation of benchmarking protocols: https://github.com/autosome-ru/motif_benchmarks [40]. All-against-all benchmarking results: https://github.com/autosome-ru/motif_benchmarking_data [43]
