# Supplementary material for: Insights gained from a comprehensive all-against-all transcription factor binding motif benchmarking study
Source: Genome Biol. 2020 May 11;21:114. doi: 10.1186/s13059-020-01996-3 (PMC7212583; doi:10.1186/s13059-020-01996-3)
Supplement: Supplementary file 1 — Additional file 1. Supplementary figures and text. [file 13059_2020_1996_MOESM1_ESM.pdf]

Insights gained from a comprehensive all-against-all transcription factor binding motif benchmarking study

Supplementary figures and description of additional files

Giovanna Ambrosini[1,2,12], Ilya Vorontsov[3,4,12], Dmitry Penzar[3,5,6], Romain Groux[1,2], Oriol Fornes[7], Daria D. Nikolaeva[5], Benoît Ballester[8], Jan Grau[9], Ivo Grosse[9,10], Vsevolod Makeev[3,6,11], Ivan Kulakovskiy[3,4,11,13], Philipp Bucher[1,2,13*]

[1] School of Life Sciences, Ecole Polytechnique Fédérale de Lausanne (EPFL), CH-1015, Lausanne, Switzerland
[2] Swiss Institute of Bioinformatics (SIB), CH-1015, Lausanne, Switzerland
[3] Vavilov Institute of General Genetics, Russian Academy of Sciences, Gubkina 3, Moscow, 119991, Russia
[4] Institute of Protein Research, Russian Academy of Sciences, Institutskaya 4, Pushchino, 142290, Russia
[5] Faculty of Bioengineering and Bioinformatics, Lomonosov Moscow State University, Leninskiye gory 1-73, Moscow, 119234, Russia
[6] Moscow Institute of Physics and Technology (State University), Institutskiy per. 9, Dolgoprudny, 141700, Russia
[7] Centre for Molecular Medicine and Therapeutics, Department of Medical Genetics, BC Children's Hospital Research Institute, University of British Columbia, Vancouver, Canada.
[8] Aix Marseille Université, INSERM, TAGC, Marseille, France.
[9] Institute of Computer Science, Martin Luther University Halle-Wittenberg, Halle (Saale), Germany.
[10] German Centre for Integrative Biodiversity Research (iDiv) Halle-Jena-Leipzig, Leipzig, Germany.
[11] Engelhardt Institute of Molecular Biology, Russian Academy of Sciences, Vavilova 32, Moscow, 119991, Russia
[12] These authors contributed equally
[13] These authors contributed equally
[*] Corresponding author. Philipp.Bucher@epfl.ch

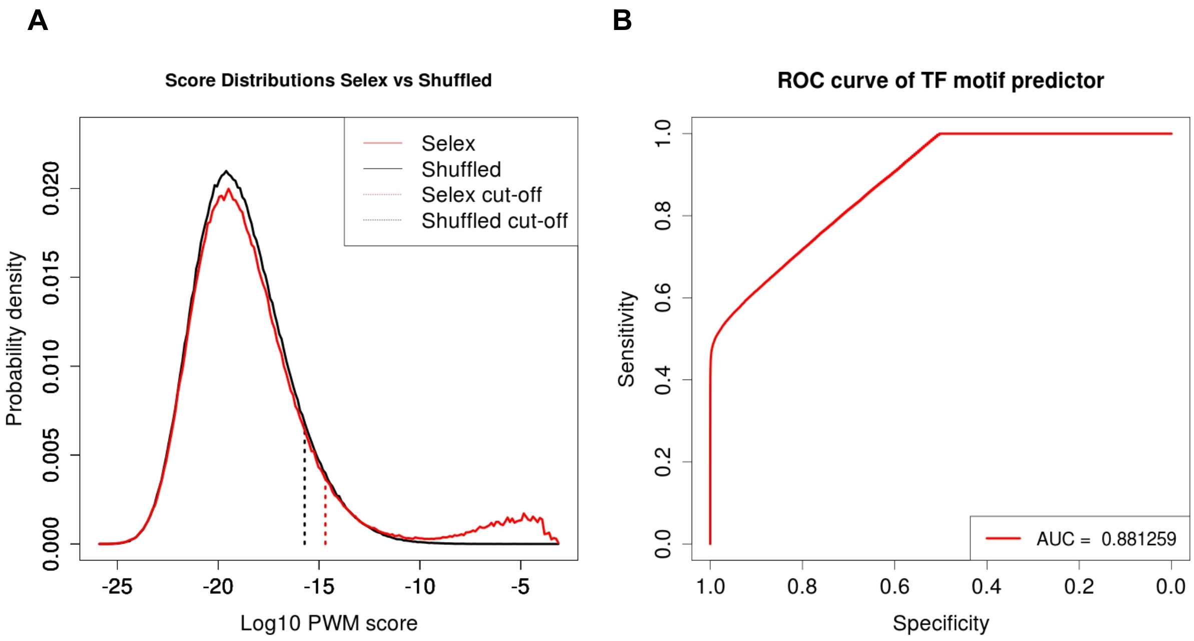

Figure S1. PWM score distribution and ROC AUC plots for an HT-SELEX library. Sum occupancy scores were computed for the combined cycles from HT-SELEX experiment IRF3_TCCTAA40NATC_AI with JASPAR matrix MA1418.1. A. Binding score distribution of the *in vitro* selected sequences (red) and shuffled control sequences (black). Note the small fraction (less than 10%) of *bona fide* protein-bound sequences represented by the tail on the right side of the score distribution. The dotted lines indicate the 10% top-score cut-offs for the positive and negative sequences. B. ROC AUC plot obtained with 10% top-scoring sequences. Setting a top-score cut-off of 50% produces a ROC AUC value of 0.572105. This figure has been produced with the PWMEval-Selex tool of the PWMTools server at https://ccg.epfl.ch/pwmtools.

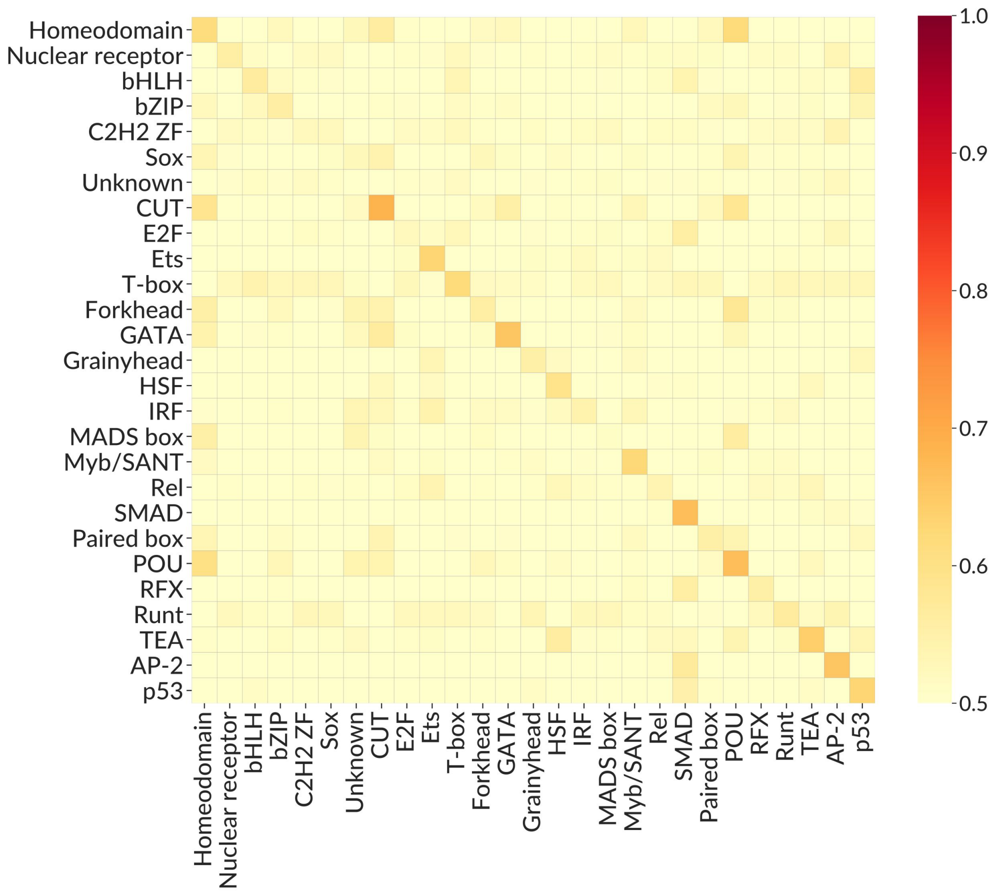

Figure S2. The average AUC ROC achieved by PWMs (rows) on particular data sets (columns). The average is taken over all binding motifs from the structural family and all experiments for all TFs from the structural family of their DNA recognition domains according to the CIS-BP TF family classification. The PWMs were benchmarked on HT-SELEX 50% data. Only families with no less than 2 PWMs, 2 ChIP-seq, and 2 SELEX data sets are shown.

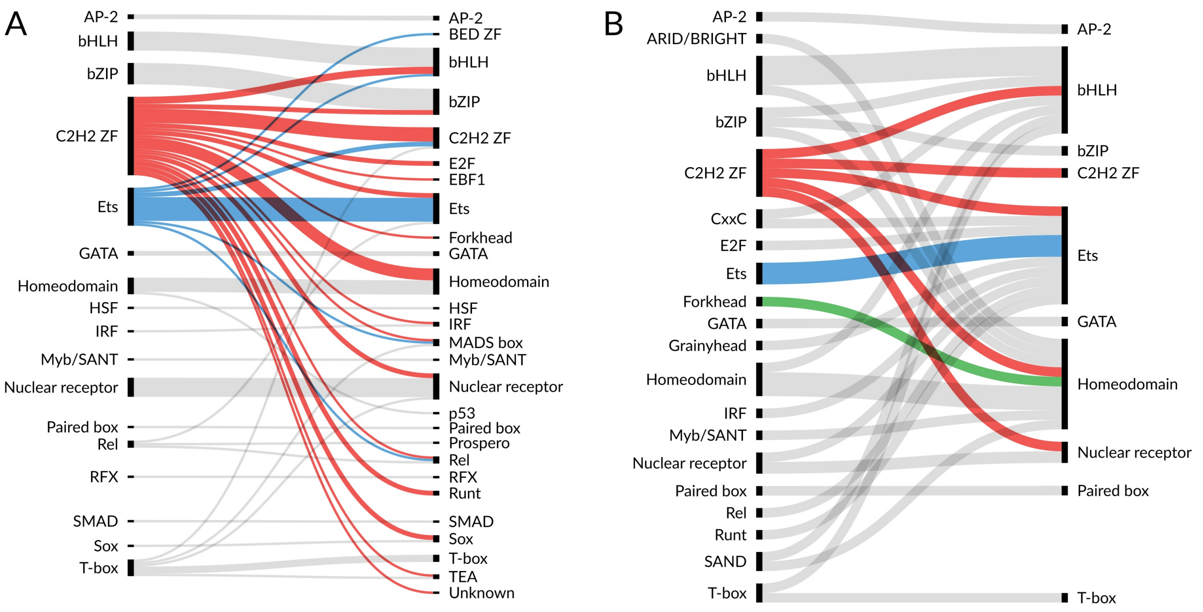

Figure S3. Alluvial plots illustrating the performance of PWMs from particular CIS-BP TF families in the HT-SELEX 50% benchmark. PWMs grouped by CIS-BP TF family are shown on the left, TFs are shown on the right. Only TFs with at least one ChIP-seq data set and one HT-SELEX data set are included. For illustration, selected motif families are highlighted with color. (A) For each TF, the PWM with the best average AUC ROC across the datasets for this TF is selected for link construction. The link width corresponds to the number of TFs. (B) For each TF, PWMs displaying the average AUC ROC of no less than 0.75 across the datasets for this TF are selected. The link width is proportional to the square root of the number of appropriate PWM-TF pairs.

TFBS motifs (matrix Ids)	HT-SELEX ROC AUC exp-1	HT-SELEX ROC AUC exp-2	PBM correlation exp-1	PBM correlation exp-2
MA0851.1 FOXJ3	0.80	0.53	0.54	0.52
FOXJ3_HUMAN.H11MO.0.A	0.76	0.53	0.53	0.50
FOXJ3_HUMAN.H11MO.1.B	0.67	0.52	0.49	0.46
M02736_2.00 FOXJ3	0.67	0.53	0.43	0.40
M02737_2.00 FOXJ3	0.81	0.53	0.59	0.59
M03046_2.00 FOXJ3	0.76	0.58	0.45	0.43
M03047_2.00 FOXJ3	0.67	0.53	0.40	0.38
M03048_2.00 FOXJ3	0.67	0.53	0.42	0.40
M04859_2.00 FOXJ3	0.78	0.53	0.52	0.51
M04860_2.00 FOXJ3	0.78	0.53	0.45	0.44
M03017_2.00 FOXJ2	0.81	0.54	0.60	0.60
M01977_2.00 FOXK1	0.80	0.55	0.67	0.65

Experiment Ids:

HT-SELEX exp-1: Foxj3_TAAACA20NCAA_AF
HT-SELEX exp-2: FOXJ3_TAGTGG20NGTT_AC
PBM exp-1: ROG18A.FoxJ3_rep1_combinatorial
PBM exp-1: ROG18A.FoxJ3_rep2_combinatorial

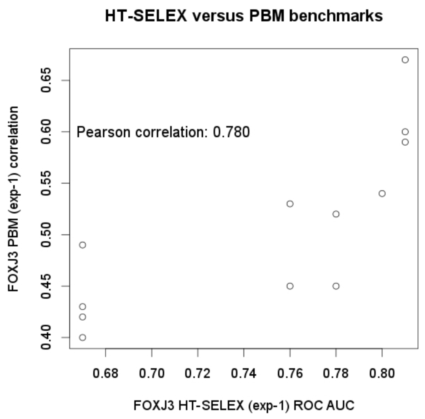

Figure S4. Comparison of ROC AUC and correlation values for 12 motif matrices tested on HT-SELEX and PBM data. The matrices were tested in parallel on two HT-SELEX and two PBM data sets for FOXJ3. The matrix collection consists of 10 matrices nominally attributed to FOXJ3 plus the overall best performing matrices for FOXJ3 on HT-SELEX and PBM data, respectively. The scatter plot illustrates the good agreement of HT-SELEX and PBM benchmarks. f the two matrices attributed to other TFs from the same family outperform all genuine FOXJ3 matrices, regardless of whether they are tested on HT-SELEX or PBM data.

NANOG_HUMAN.H11MO.0.A SOX2-POU2F1 motif from CAP-SELEX

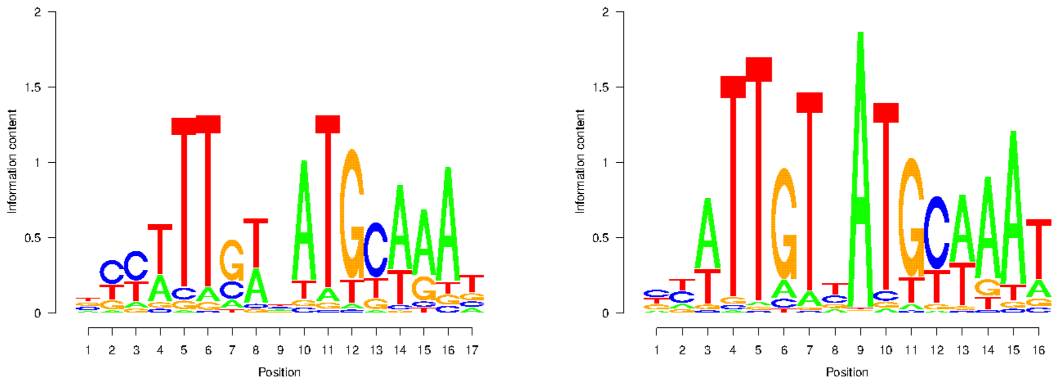

Figure S5. Confirmation of an indirect binding mode for a NANOG matrix with CAP-SELEX. All-against-all benchmarking of NANOG_HUMAN.H11MO.0.A using *in vivo* ChIP-seq data suggests that this matrix represents a composite DNA motif consisting of a SOX2 and a POU5F1 binding site (Table 3 of main text). This, in turn, supports the hypothesis that the Nanog protein can be recruited indirectly to target sites through protein-protein interaction with SOX2-POU5F1 heterodimers bound to DNA. CAP-SELEX is a technique that allows for characterization of the DNA specificity of TF-heterodimers (Jolma et al. 2015, Nature 527:384). The sequence logo on the right side shows the motif extracted from a CAP-SELEX experiment targeted at SOX2-POUF2F1 dimers, published in the supplemental material of the CAP-SELEX paper. Assuming that POUF2F1 and POUF5F1 have similar binding specificity, the strong resemblance between the two motifs supports the indirect binding mode hypothesis for Nanog. This example also underscores the usefulness of CAP-SELEX for the interpretation of *in vivo* binding experiments.

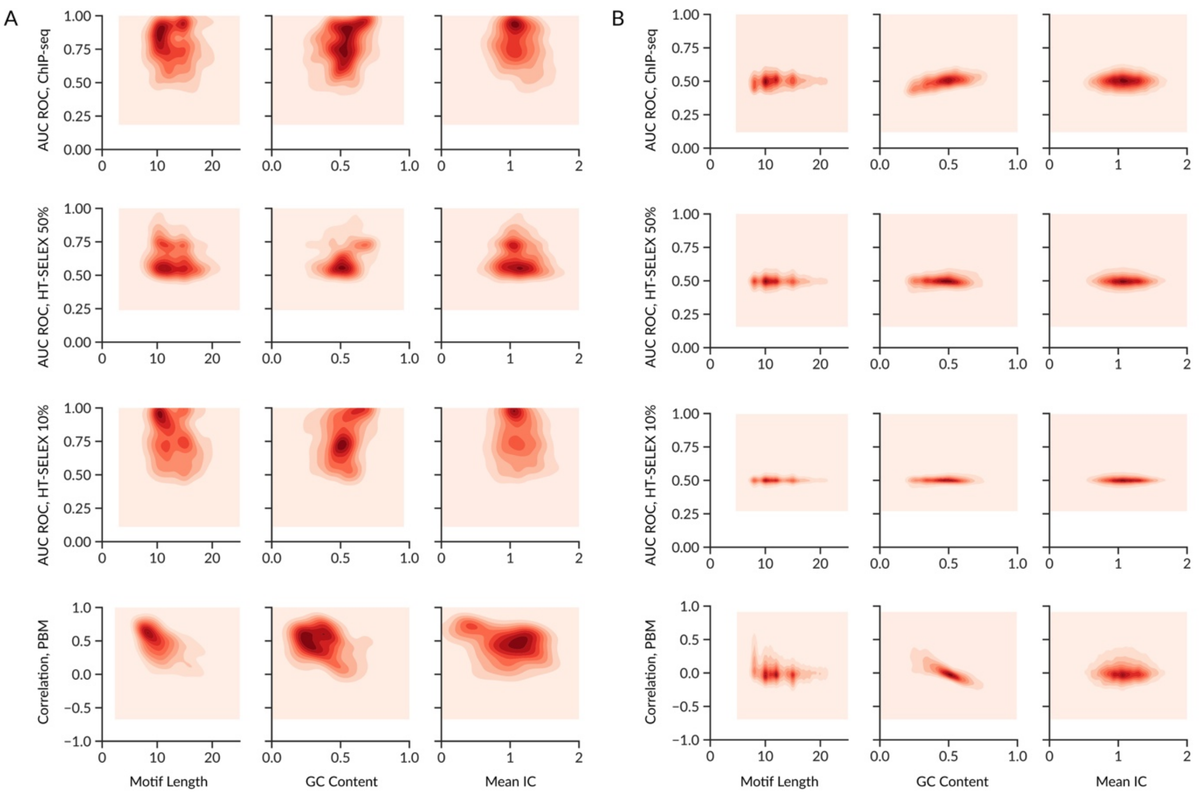

C

Benchmark	Same TF combinations			All combinations		
	Motif Length	GC Content	Mean IC	Motif Length	GC Content	Mean IC
**AUC ROC, ChIP-seq**	-0.002 (R^2=0.001)	**0.285 (R^2=0.054)**	**-0.074 (R^2=0.016)**	**-0.001 (R^2=0.001)**	**0.163 (R^2=0.123)**	0 (R^2=0)
**AUC ROC, HT-SELEX 50%**	**-0.006 (R^2=0.026)**	**0.170 (R^2=0.032)**	-0.034 (R^2=0.005)	0 (R^2=0)	**0.030 (R^2=0.009)**	**-0.001 (R^2=0)**
**AUC ROC, HT-SELEX 10%**	**-0.006 (R^2=0.015)**	**0.308 (R^2=0.053)**	-0.057 (R^2=0.008)	**0 (R^2=0)**	**0.060 (R^2=0.013)**	**-0.004 (R^2=0)**
**Correlation, PBM**	**-0.032 (R^2=0.217)**	**-0.612 (R^2=0.169)**	**-0.135 (R^2=0.048)**	**-0.009 (R^2=0.047)**	**-0.824 (R^2=0.479)**	**-0.041 (R^2=0.005)**

Figure S6. Kernel density plots illustrating relations between basic motif features (motif length, GC content, mean information content) and motif performance in ChIP-seq, HT-SELEX 10%, HT-SELEX 50%, and PBM benchmarks. Each point underlying the density plot corresponds to a single PWM of a particular TF evaluated on a particular data set. AUC ROC (for ChIP-seq and SELEX) and Pearson correlation coefficients (for PBM) are calculated by averaging corresponding values over all datasets of that TF. Mean GC-content is estimated as the total fraction of GC nucleotides across all positions of the position frequency matrix. Mean information content is an average over positions of (2 minus the entropy of nucleotide distribution). (A) PWMs and experimental data sets associated with the same TFs. (B) All possible combinations of PWMs and experimental data sets. (C) Slope and R^2 values of trend lines. Cells with P-values of Pearson correlation between motif features and prediction performance measures of less than 0.001 are presented in bold.

Description of additional files

Additional files 2-5: These files contain interactive supplementary figures. Dimensionality reduction with t-SNE is applied to PWMs' performance at ChIP-seq (file 2), HT-SELEX 10% (file 3), HT-SELEX 50% (file 4), and PBM data (file 5). Each point corresponds to a PWM. Coloring schemes correspond to TFClass classes (level 2), TFClass families (level 3), CIS-BP families, motif experimental source data, and motif collections.

Additional file 6: Detailed annotation including ID mapping and family classification of the human transcription factors and corresponding motifs of JASPAR/HOCOMOCO/CIS-BP used in the study.

Additional file 7: List of PBM data sets from UniPROBE used in this study.

Description of data and code repositories

The code and data are available under MIT license. Benchmarking protocols have been containerized using docker to make them easily reusable, and published as Zenodo repository [doi:10.5281/zenodo.3695374] *.

Performance measures and benchmarking results are also published as [doi:10.5281/zenodo.3702150] **.

The data repository contains the link to the collection of supplementary scripts used to prepare the Figures and Tables for the publication [https://github.com/autosome-ru/motif_benchmarking_paper]. These supplementary scripts require manual intervention (but may potentially be useful for understanding the analysis).

* Philipp Bucher, Giovanna Ambrosini. [Ilya Vorontsov, Dmitry Penzar, Romain Groux, Oriol Fornes, Daria Nikolaeva, Benoit Ballester, Jan Grau, Ivo Grosse, Vsevolod Makeev, Ivan Kulakovskiy] Benchmarks for the paper "Insights gained from a comprehensive all-against-all transcription factor binding motif benchmarking study". https://github.com/autosome-ru/motif_benchmarks. doi:10.5281/zenodo.3695374 (2020)

** Philipp Bucher, Giovanna Ambrosini. [Ilya Vorontsov, Dmitry Penzar, Romain Groux, Oriol Fornes, Daria Nikolaeva, Benoit Ballester, Jan Grau, Ivo Grosse, Vsevolod Makeev, Ivan Kulakovskiy] Data for the paper "Insights gained from a comprehensive all-against-all transcription factor binding motif benchmarking study". https://github.com/autosome-ru/motif_benchmarking_data. doi:10.5281/zenodo.3702150 (2020)
