# Supplementary material for: Insights gained from a comprehensive all-against-all transcription factor binding motif benchmarking study
Source: Genome Biol. 2020 May 11;21:114. doi: 10.1186/s13059-020-01996-3 (PMC7212583; doi:10.1186/s13059-020-01996-3)
Supplement: Supplementary file 4 — Additional file 4. Interactive t-SNE plots for HT-SELEX (cut-off 50%). [file 13059_2020_1996_MOESM4_ESM.html]

### Additional file. Dimensionality reduction with t-SNE applied to PWMs performance at HT-SELEX 50% data. Each point corresponds to a PWM. Coloring schemes correspond to TFClass classes (level 2), TFClass families (level 3), CIS-BP families, Motif experimental source data, and Motif collections.
