# Supplementary material for: Insights gained from a comprehensive all-against-all transcription factor binding motif benchmarking study
Source: Genome Biol. 2020 May 11;21:114. doi: 10.1186/s13059-020-01996-3 (PMC7212583; doi:10.1186/s13059-020-01996-3)
Supplement: Supplementary file 8 — Additional file 8. Review history. [file 13059_2020_1996_MOESM8_ESM.docx]

Review History

**First round of review**

**Reviewer 1**

**Are you able to assess all statistics in the manuscript, including the appropriateness of statistical tests used?**

Yes, and I have assessed the statistics in my report.

**Comments to author:**

Ambrosini et al. present a comprehensive test of many publicly available binding models on high-throughput experimental data. The study identified that many TFs may provide better binding prediction of TFs in the same protein family than their own-specific binding models. Moreover, it identified several cases of protein cooperativity.

While the study is interesting, well-written and comprehensive, it is difficult to judge it as it reports intermediate results. As some of our major comments, and future directions mentioned in the discussion, the study seems incomplete. Thus, we believe that more work should be done before the study can be published.

Major comments:
1.     Regarding the PWM source database. First, cis-bp is the most recent comprehensive database of PWMs and should be included in the tests. Second, PWMs should be excluded if their parameters were trained on the same test data (ChIP-seq or HT-SELEX), otherwise this is a circular test.
2.     The HT-SELEX analysis is problematic and unclear. First, the HT-SELEX negative set is synthetic and unrealistic. As done in Dror et al. GR 2015, the input is a better negative set. Second, it is unclear what are the 10% and 50% top sequences - are they ranked by occurrence in HT-SELEX data or by PWM score? It is not clear why this is necessary at all, and why not all sequences are used. The high scores (0.99) in Table 3 are an indication of this benchmark being too easy to classify.
3.     Since both tests only considered classification, an excellent benchmark to use is testing on protein binding microarrays. They would provide the ability to test whether PWMs can predict binding intensity, as a common measure of accuracy is Pearson correlation between predicted and measured binding intensities. The UniPROBE database already hosts more than 1000 experiments. It is critical to see if the results and conclusions reached on ChIP-seq and HT-SELEX data are reproduced on PBM data.

Minor comments:
1.     Figure 1 - we recommend using labels for sub-panels, and shortening the caption.
2.     Methods, "pseudo-probability" should be referred to as Laplace correction.
3.     HT-SELEX sets were augmented with additional sequencing by Lin et al. MSB 2017. The sequencing from that study should be downloaded and added.
4.     We recommend visualizing Tables 1 and 2 as bar plots with numbers on top of the bars.
5.     Figures 4 and 5 are difficult to read, as well as Figure 2 labels. One suggestion is to remove the 'related factors' suffix for each protein family label.
6.     It would be interesting to inspect what are the common characteristic of 'best performing motifs' - are they shorter or longer than most motif? Do they have a unique GC-content? Information content that is higher or lower?
7.     Table 3 should be visualized as a bar plot. Each experiment should be a bar. Moreover, the sequence logo of ELK1 and some of the other proteins should be visualized.
8.     Recently, Jolma et al. published a paper in Nature 2016 on TF pairs. The results of the last Results subsection may be validated by the experiments in this dataset.
9.     Tables 4 and 5 are difficult to read. The numbers should be converted to a bar plot. In addition, the labels should be only the protein name, otherwise there is too much irrelevant text in the plot.
10.     For ChIP-seq it is also possible to test free DNA regions compared to nucleosome-occupied regions and compute an AUPR score as there are many more negatives. If it is too difficult to perform, this option should be at least mentioned under future directions.

Typos:
1.     Abstract: last sentence, model - > models.
2.     P.3 l. 14: remove second "the".
3.     P.4 l. 56, Information -> information OR Information Content.
4.     P.5, l. 34: form -> a form.
5.     P. 10, l. 1: imported -> downloaded.
6.     P. 10, l. 30 - aren't HT-SELEX experiments of length 14-40? (14, 20, 30, 40).
7.     Table 1 reports 547 HT-SELEX experiments, while the number 548 appears somewhere else in the manuscript.
8.     Table 1 caption: Given -> Given in.
9.     P. 16, l. 38 - two spaces.
10.     P. 19, l. 42: missing closing bracket "]".
11.     Table 5 caption: ">0.75" should be removed.
12.     Both 'dataset' and 'data set' appear in the text. There should be consistence.

**Reviewer 2**

**Are you able to assess all statistics in the manuscript, including the appropriateness of statistical tests used?**

There are no statistics in the manuscript.

**Comments to author:**

With the increasing number of TF binding motifs and experimental data for the TFs, it is interesting to update our knowledge about TF and TF binding sites based a large scale all TFBS motifs against all TF binging experimental data. The authors present here a comparison of more than 1000 motifs against more than 2000 ChiP-seq experiment results and more than 500 HT-SELEX experiment datasets. Results showed that similar binding specificity across many but not all TF families. A non-redundant motif set were created with about 10 times smaller than all motifs available. This is a novel evaluation of the large amount of existing TF motifs and experimental results. It expanded our understanding about TF binding. Some issues are listed below.

Major revisions:

1. All tests were based on the TFClass dataset, though the reliability of using this gold standard dataset was not accessed. It is better to include some evaluation of the accuracy of the TF motif and TF binding experimental data comparison methods.    
2. Font sizes of Fig 4 and Fig5 were too small. A better way with larger font size should be presented.

**Authors Response**

**Point-by-point responses to the reviewers’ comments:**

Reviewer #1: Ambrosini et al. present a comprehensive test of many publicly available binding models on high-throughput experimental data. The study identified that many TFs may provide better binding prediction of TFs in the same protein family than their own-specific binding models. Moreover, it identified several cases of protein cooperativity.

While the study is interesting, well-written and comprehensive, it is difficult to judge it as it reports intermediate results. As some of our major comments, and future directions

mentioned in the discussion, the study seems incomplete. Thus, we believe that more work should be done before the study can be published.

*In response to the reviewer's concern about incompleteness, we massively expanded our all-against-all benchmarking study in the following ways:*

*We have defined a new benchmarking protocol for protein-binding microarray (PBM) data.*

*We have added 100 PBM experiments for human transcription factors to the total database of test data sets.*

*We have added 3622 TFBS motif matrices from CIS-BP to the previously used 1350 matrices from JASPAR and HOCOMOCO.*

*We have added the new HT-SELEX data from Yang et al. 2017 to the previously used SELEX data from Jolma et al. 2013. This has increased the total volume of the HT-SELEX data by roughly a factor of 10.*

*We have recomputed all performance values for the extended SELEX data with the old matrix collection.*

*We have benchmarked the old matrix collection with the new PBM data sets.*

*We have benchmarked the new matrices from CIS-BP on all ChIP-seq, HT-SELEX, and PBM data sets.*

*All Figures and Tables in the old manuscript were redone using the new performance values.*

*In summary, the total number of performance values computed (and made public to researchers) has increased from about 4 million to about 15 million.*

Major comments:

Regarding the PWM source database. First, cis-bp is the most recent comprehensive database of PWMs and should be included in the tests.

*See* *above.*

Second, PWMs should be excluded if their parameters were trained on the same test data (ChIP-seq or HT-SELEX), otherwise this is a circular test.

*We agree in principle, but this is hardly feasible because the matrices from public resources are not systematically linked to the particular experiments from which they were derived. Even CIS-BP matrices annotated as being derived from HT-SELEX or PBM cannot be traced to individual experiments when multiple experiments have been carried out for the same TF. However, we agree that the problem of over-fitting needs to be discussed although we don't think that the problem is so critical for this study. For most TFs, there are multiple test sets in our collection. Users of our benchmarking results can thus assess the quality of a given matrix based on the robustness of its performance across multiple data sets. Furthermore, the majority of best performing matrices for a given experiment are assigned to a different TF in the source databases. These best-performing matrices were obviously derived from different experiments, indicating that over-fitting is not a major source of bias in this study.*

*We have explained this in similar terms in the Discussion Section of the revised manuscript.*

The HT-SELEX analysis is problematic and unclear. First, the HT-SELEX negative set is synthetic and unrealistic. As done in Dror et al. GR 2015, the input is a better negative set.

*We have recomputed all performance values using the input libraries as negative*

*controls. All Figures and Tables now reflect the new performance values. The primary results changed very little and conclusions of the study remain the same.*

Second, it is unclear what are the 10% and 50% top sequences - are they ranked by occurrence in HT-SELEX data or by PWM score?

*They are ranked by PWM score. As mentioned in the text, we eliminated duplicates from the SELEX libraries (assuming they are copies of the same founder molecules produced by PCR). Therefore, we cannot use re-occurrence as a ranking criterion. We realize that the benchmarking protocol was not well described in the previous version of the manuscript. We have changed the description as follows:*

*"Protocol for HT-SELEX data:This protocol is applicable to all flavors of SELEX, which enrich a random pool of DNA oligonucleotides for sequences with high affinity to a particular DNA-binding protein of interest. The data from such an experiment consist of a library of DNA sequences of constant length (typically 15-40 bp). Current high-throughput SELEX technologies produce millions of sequences per experiment. As with the ChIP-seq peak-based evaluation method, we need a negative sequence set, which could be obtained by shuffling the positive sequences. Alternatively, sequences from the input library used in the protein-DNA binding reaction may be available for this purpose. From this point on, we proceed in a similar way as with the ChIP-seq peak lists. We first compute sum occupancy scores for all sequences in both libraries. However, because SELEX libraries are often only weakly enriched with true binding sequences (Figure S1, Additional file 1), we take only a top percentile of the positive and negative scores (e.g. the top 10%) for ROC AUC value computation. Importantly, before PWM scoring we extend the random insert sequences obtained from the sequence repository with the primer and barcode sequences that were present (and thus accessible to proteins) during the SELEX experiments.*

*and we added supplementary Figure S1 illustrating the principle of the method.*

It is not clear why this is necessary at all, and why not all sequences are used. The high scores (0.99) in Table 3 are an indication of this benchmark being too easy to classify.

*We agree that in this case, the benchmark is too easy, at least for the top-ranked experiments. We thus added the performance values obtained with the top 50% scores to Table 1 (former Table 3) for comparison.*

Since both tests only considered classification, an excellent benchmark to use is testing on protein binding microarrays. They would provide the ability to test whether PWMs can predict binding intensity, as a common measure of accuracy is Pearson correlation between predicted and measured binding intensities. The UniPROBE database already hosts more than 1000 experiments. It is critical to see if the results and conclusions reached on ChIP-seq and HT-SELEX data are reproduced on PBM data.

*We have benchmarked all matrices on UniPROBE PBM data.*

*A supplementary Figure (S4) shows the congruence between performance values obtained for set SELEX and PBM for a collection of different matrices for the same factor. The following text has been added to the manuscript:*

*" We were nevertheless wondering whether matrices performing well in a qualitative, classification-based test, were able to predict protein-affinity determined by a quantitative protein-binding assay. Examining several cases, were a TF has been assayed by at least two different techniques, and multiple matrices were available for the same TF, led us to the conclusions that this is indeed the case. An example is presented in Figure S4 (Additional file 1) where the ROC AUC values of 12 different PWMs for FOXJ3 obtained on SELEX-data were compared to corresponding correlation coefficients obtained on PBM data. The two number series are highly concordant with a correlation coefficient of 0.84."*

Minor comments:

Figure 1 - we recommend using labels for sub-panels, and shortening the caption.

*Done.*

Methods, "pseudo-probability" should be referred to as Laplace correction.

*After discussion among all authors we agreed to disagree with the reviewer. We think that "pseudo-probabilities" would be understandable to a larger number of prospective readers than "Laplace correction". Finally, we opted for the following formulation:*

*" The original matrices were slightly modified by adding a correction term .."*

HT-SELEX sets were augmented with additional sequencing by Lin et al. MSB 2017. The sequencing from that study should be downloaded and added.

*Done, as pointed out at the beginning.*

We recommend visualizing Tables 1 and 2 as bar plots with numbers on top of the bars*.*

*Done.*

Figures 4 and 5 are difficult to read, as well as Figure 2 labels. One suggestion is to remove the 'related factors' suffix for each protein family label.

*We now use the CIS-BP TF family classification, which allowed us to simplify the figures.*

It would be interesting to inspect what are the common characteristic of 'best performing motifs' - are they shorter or longer than most motif? Do they have a unique GC-content? Information content that is higher or lower?

*We tested several properties (GC-content, motif length (PWM width), average information content) and did not find any clear characteristic linked to benchmarking performance. The results are presented as kernel density plots in the Supplementary Figure S6.*

Table 3 should be visualized as a bar plot. Each experiment should be a bar. Moreover, the sequence logo of ELK1 and some of the other proteins should be visualized.

*We prefer the Table format. Bar plots are appropriate for counts or percentages, quantities for which zero means "nothing". ROC AUC performance measures do not fall into this category.*

*We also didn't follow the advice to add "sequence logos of some of the other factors", because logos belong to matrices not factors. However, we included the logo for the matrix under consideration.*

Recently, Jolma et al. published a paper in Nature 2016 on TF pairs. The results of the last Results subsection may be validated by the experiments in this dataset.

*Good suggestion by the reviewer. We indeed were able to validate a speculated composite binding motif of a matrix from Table 3 (former Table 5) with a TF pair motif from Jolma et al. 2015. This new finding has been reported as follows in the revised version of the manuscript.*

*" Recently, a modified HT-SELEX protocol has been published ((34), enabling the characterization of TF heterodimers. We were wondering whether the hypothesized indirect binding target of Nanog mediated by a SOX2-POU5F1 dimer was supported by new data generated with this technology. This is indeed the case. The authors of the above-cited paper were able to extract a motif for a closely related complex, a SOX2-POU2F1 TF pair (Figure S5, Additional file 1), which closely resembles the sequence logo shown on the left side of Table 3."*

Tables 4 and 5 are difficult to read. The numbers should be converted to a bar plot. In addition, the labels should be only the protein name, otherwise there is too much irrelevant text in the plot.

*For reasons explained above, we kept the Table format (new Tables 2 and 3). We also disagree that labels should only be protein names. There are multiple experiments for the same protein. Leaving out experiments name would mean publishing irreproducible numbers.*

For ChIP-seq it is also possible to test free DNA regions compared to nucleosome-occupied regions and compute an AUPR score as there are many more negatives. If it is too difficult to perform, this option should be at least mentioned under future directions.

*We have addressed this discussion point as follows in the revised manuscript.*

*" For the ChIP-seq peak-list based method, there may be better choices for the negative control set. For instance, one could try to match positive and negative genomic regions in terms of properties such as chromatin accessibility or nucleosome occupancy (if data are available for the corresponding cell type)."*

Typos:

Abstract: last sentence, model - > models.

P.3 l. 14: remove second "the".

P.4 l. 56, Information -> information OR Information Content.

P.5, l. 34: form -> a form.

P. 10, l. 1: imported -> downloaded.

P. 10, l. 30 - aren't HT-SELEX experiments of length 14-40? (14, 20, 30, 40).

Table 1 reports 547 HT-SELEX experiments, while the number 548 appears somewhere else in the manuscript.

Table 1 caption: Given -> Given in.

P. 16, l. 38 - two spaces.

P. 19, l. 42: missing closing bracket "]".

Table 5 caption: ">0.75" should be removed.

Both 'dataset' and 'data set' appear in the text. There should be
consistence.

*All corrected.*

Reviewer #2: With the increasing number of TF binding motifs and experimental data for the TFs, it is interesting to update our knowledge about TF and TF binding sites based a large scale all TFBS motifs against all TF binging experimental data. The authors present here a comparison of more than 1000 motifs against more than 2000 ChiP-seq experiment results and more than 500 HT-SELEX experiment datasets. Results showed that similar binding specificity across many but not all TF families. A non-redundant motif set were created with about 10 times smaller than all motifs available. This is a novel evaluation of the large amount of existing TF motifs and experimental results. It expanded our understanding about TF binding. Some issues are listed below.

Major revisions:

1. All tests were based on the TFClass dataset, though the reliability of using this gold standard dataset was not accessed. It is better to include some evaluation of the accuracy of the TF motif and TF binding experimental data comparison methods.

*We are not sure whether we correctly understand the reviewer's request. We reacted to this comment by using a second TF domain and family classification scheme (CIS-BP TF family annotation) in our work. Some results were produced in parallel with the two classifications (see The Additional files 2-5 with interactive plots). Overall the results are congruent, as expected, because the classification systems themselves largely agree with each other.*

.

2. Font sizes of Fig 4 and Fig5 were too small. A better way with larger font size should be presented.

*Taken this into account, these Figures have been redone.*

**Second round of review**

**Reviewer 1**

Major comments:
1.     HT-SELEX data cover both human and mouse TFs and was used with no filtering. But, only human PBM data was used. We recommend using PBM mouse data in addition to human, as it would add more data to the benchmarks and be consistent with the use of HT-SELEX data.
2.     Following point (1), in almost all analyses no results are reported for PBM benchmarking (e.g. Table 1). It might be due to few datasets, but by increasing it using mouse TFs, all PBM benchmarks should be reported, as for ChIP-seq and HT-SELEX.
3.     P. 28, l. 16 – the Discussion only talks about two different benchmarking protocols, while three were used. The PBM benchmark should also be made publicly available through a web interface and a docker image.

Minor comments:
1.     One Word comment (Figure 7) was left in the manuscript, and the response to reviewers has modifications under Word Track Changes.
2.     P. 10, l. 21: HT-SELEX lengths of 15-40 is wrong as the data form Taipale’s lab has lengths 14, 20, 30 and 40.
3.     Figure S1 (and other figures): when there are multiple sub-panels, mark them in English letters, i.e. A and B, and refer to (A) and (B) in the caption (instead of right and left side, for example).
4.     P. 11, l. 11: there was no reasoning for the use of log sum occupancy score instead of sum occupancy score. A reason should be given.
5.     P. 13, l. 25 – AUC ROC is not used for PBM. PBM data and the Pearson correlation achieved on it should be considered in this evaluation of globally best performing matrix.
6.     Figure 4 should include PBM benchmarking.
7.     Figure 4 plots have no axis ticks or axes labels.
8.     Figure 4 symbols (o, x) should be in a legend, not the caption.
9.     Figure 5A, S3A: all links seem to be in the same width. Is there an error? If it’s only a single TF per link, then the caption should not state “the width is the number of TFs”.
10.     Figures 5,6: it is unclear what led to coloring some families and not the others. The caption only states: “Selected TF families are highlighted with color”.
11.     P. 18, l. 28-30: it is unclear why the fact that Pearson correlation is used prohibits choosing the best performing matrix.
12.     Tables 1, 2, 3 and S4 are badly formatted – they are textual as if taken from a unix terminal. Moreover, in S4, it should be note that PBM uses Pearson correlation. In addition, it is enough to present two digits after the decimal point. The tables are very difficult to read (they should be visualized as bar plots), and the textual formatting adds to the difficulty.
13.     P. 28, l. 22: there is a claim that including barcode sequences improves AUC ROC for HT-SELEX prediction, but no data was shown for it.
14.     P. 31, l. 35: the description is unclear: “20 bp pair sequences” – first, the sentence is grammatically incorrect. Second, the primers presented below are longer than 20 bp. We suggest revising to “after flanking with barcode and primers on both sides, sequences were truncated to include only the 20bp adjacent to the random insert on each flank”.
15.     P. 32: it should be mentioned that only human PBM datasets were used, as this is the Methods section reporting how data was downloaded.

Typos:
1.     P. 5, l. 50 “see (17) and (17)” – it should be two different citations.
2.     P. 11, l. 52: Weirauch et al. 2014 in parenthesis and (13) is cited. There are other cases in the text with textual as opposed to numerical citation. These should be correct for one of the citation formats.
3.     Both “data set” and “dataset” are in the text – this was not corrected, despite the authors’ claim in their response.
4.     Both “Ets” and “ETS” are used in the text and figures. Be consistent.
5.     P. 21, l. 16: “A single PWMs” -> “A single PWM”.
6.     P. 24, l. 23: what is “(Stark and (22)”?
7.     P. 25, l. 12: Extra open parenthesis.
8.     “Nanog” and “NANOG” both appear in the text and figures. Be consistent.
9.     Both “in vivo” and “in vivo” appear in the text. Be consistent.
10.     P. 27, l. 37: i is not italicized in “in vitro”.
11.     P. 29, l. 22: add a comma after Thus.

**Reviewer 2**

A new dataset was added to evaluation and the figure quality has been improved. All my concerns have been addressed.

**Authors Response**

**Point-by-point responses to the reviewers’ comments:**

HT-SELEX data cover both human and mouse TFs and was used with no filtering. But, only human PBM data was used. We recommend using PBM mouse data in addition to human, as it would add more data to the benchmarks and be consistent with the use of HT-SELEX data.

*Done. In addition to human data PBM mouse data has been processed. The new data are available now from:* [*ftp://ccg.vital-it.ch/pwmtools/benchmarking/uniprobe2**](ftps://ccg.vital-it.ch/pwmtools/benchmarking/uniprobe2*)

Following point (1), in almost all analyses no results are reported for PBM benchmarking (e.g. Table 1). It might be due to few datasets, but by increasing it using mouse TFs, all PBM benchmarks should be reported, as for ChIP-seq and HT-SELEX.

*PBM benchmarking has been conducted at the same scale as ChIP-seq and HT-SELEX. In particular Figures 2, 4, 5, 6 and S6, as well as Tables 1-3 now include the results from PBM benchmarking. The text of the paper has been updated accordingly.*

P. 28, l. 16 - the Discussion only talks about two different benchmarking protocols, while three were used. The PBM benchmark should also be made publicly available through a web interface and a docker image

*We have dockerized the PBM protocol, and it is available in the same repository as those for SELEX and ChIP-seq. We plan to offer PBM benchmarking also from our web server, but were not able to achieve this during revision time. One complication is that the license agreement governing the usage of data from UniPROBE doesn't allow us to put their data behind our web server. We could offer a web service where users could upload appropriately formatted PBM data (possibly downloaded from UniPROBE) or try to negotiate permission to host their data on our web server. We will pursue both options.*

Minor comments:

One Word comment (Figure 7) was left in the manuscript, and the response to reviewers has modifications under Word Track Changes.

*The comment to Figure 7 has been removed.*

P. 10, l. 21: HT-SELEX lengths of 15-40 is wrong as the data form Taipale's lab has lengths 14, 20, 30 and 40.

*Corrected.*

Figure S1 (and other figures): when there are multiple sub-panels, mark them in English letters, i.e. A and B, and refer to (A) and (B) in the caption (instead of right and left side, for example).

*Done*

P. 11, l. 11: there was no reasoning for the use of log sum occupancy score instead of sum occupancy score. A reason should be given.

*This was an error in the text:*

*"Pearson correlation values between normalized probe intensities and log*

*sum-occupancy scores (see Methods)"*

*This was corrected to:*

*"Pearson correlation values between normalized log probe intensities and*

*log sum-occupancy scores (see Methods)"*

*in accordance with the text in the Methods Section.*

P. 13, l. 25 - AUC ROC is not used for PBM. PBM data and the Pearson correlation achieved on it should be considered in this evaluation of globally best performing matrix.

*Done, but this required a major change on the optimality criterion. AUC ROC and Pearson correlation coefficients are not directly comparable (for instance what is better, AUC ROC 0.9 or Pearson correlation 0.6? – we simply cannot say). In the previous analysis, the globally best performing matrix was chosen as the one, which achieves the highest AUC ROC value over all experiments for the same gene. Now we use a so-called aggregate rank score defined in the Methods Section as follows:*

*Aggregate rank score and identification of the best performing matrix for a gene: All rows corresponding to a given gene were extracted from a complete table containing performance measures (ROC AUC or correlation coefficients) for combinations of experiments (rows) and TF motif matrices (columns). The numbers in a given row were first converted into ranks. The overall performance of a matrix for multiple experiments for the same gene was then computed as the geometric mean over the ranks. This score is referred to as the "aggregate rank score" elsewhere in the text.*

Figure 4 should include PBM benchmarking.

*We have added a new panel that illustrates the results of the updated PBM benchmarking.*

Figure 4 plots have no axis ticks or axes labels.

*We have added axes labels as suggested. We have not added axis ticks as t-SNE plots as actual values of t-SNE components are not informative (see e.g. t-SNE guidelines of Kobak and Berens,* [*https://doi.org/10.1038/s41467-019-13056-x).*](https://doi.org/10.1038/s41467-019-13056-x).)

Figure 4 symbols (o, x) should be in a legend, not the caption.

*Corrected as suggested.*

Figure 5A, S3A: all links seem to be in the same width. Is there an error? If it's only a single TF per link, then the caption should not state "the width is the number of TFs".

*We thank the reviewer for marking an important flaw. Indeed, the link width was incorrect. We have updated the respective figures and captions in the revised version.*

Figures 5,6: it is unclear what led to coloring some families and not the others. The caption only states: "Selected TF families are highlighted with color".

*Three well-studied TF families were selected as illustrative examples. We have updated the figure caption accordingly.*

P. 18, l. 28-30: it is unclear why the fact that Pearson correlation is used prohibits choosing the best performing matrix.

*See explanations under minor revision point 5. The text concerned by this remark has been deleted.*

Tables 1, 2, 3 and S4 are badly formatted - they are textual as if taken from a unix terminal. Moreover, in S4, it should be note that PBM uses Pearson correlation. In addition, it is enough to present two digits after the decimal point. The tables are very difficult to read (they should be visualized as bar plots), and the textual formatting adds to the difficulty.

*We have reduced the numbers in the Tables to two digits after the decimal point. We have explained the reasons why we are against bar plots in our response letter accompanying the first revision:*

*We prefer the Table format. Bar plots are appropriate for counts or percentages, quantities for which zero means "nothing". ROC AUC performance measures do not fall into this category.*

P. 28, l. 22: there is a claim that including barcode sequences improves AUC ROC for HT-SELEX prediction, but no data was shown for it.

*We do not quantitatively justify this claim but mentioned an example to better explain why including barcodes and primers matters:*

*An important novelty of our approach is that we include primer and barcode sequences in the evaluation of HT-SELEX data, in order to account for overlapping binding sites. For instance, the barcode of the HT-SELEX library named "ELK1_TCGGAA20NAGT" provides the 3'terminal ATG of the ELK1 motif (CCGGAATG), and as a consequence contains many inserts ending with TCGGA. These binding sites are missed if only inserts are scanned.*

P. 31, l. 35: the description is unclear: "20 bp pair sequences" - first, the sentence is grammatically incorrect. Second, the primers presented below are longer than 20 bp. We suggest revising to "after flanking with barcode and primers on both sides, sequences were truncated to include only the 20bp adjacent to the random insert on each flank".

*The text was modified as suggested by the reviewer.*

P. 32: it should be mentioned that only human PBM datasets were used, as this is the Methods section reporting how data was downloaded.

*Mouse PBM data are now use as well and the text has been modified accordingly.*

Typos:

P. 5, l. 50 "see (17) and (17)" - it should be two different citations.

*Done. Second reference is Hu et al. 2005.*

P. 11, l. 52: Weirauch et al. 2014 in parenthesis and (13) is cited. There are other cases in the text with textual as opposed to numerical citation. These should be correct for one of the citation formats.

*Done (duplicated citation removed).*

Both "data set" and "dataset" are in the text - this was not corrected, despite the authors' claim in their response.

*Done. We now consistently used "data set"*

Both "Ets" and "ETS" are used in the text and figures. Be consistent.

*"Done. We now consistently use ETS except in two cases: a) when we refer to the family 3.5.2 "Ets-related" from TFclass b) when we refer to the " Ets/IRF composite element" defined in (Tamura et al. 2005)."*

P. 21, l. 16: "A single PWMs" -> "A single PWM".

*Done.*

P. 24, l. 23: what is "(Stark and (22)"?

*Done. Has become just one reference (Stark and Darnell, 2012).*

P. 25, l. 12: Extra open parenthesis.

*The parenthesis has been removed.*

"Nanog" and "NANOG" both appear in the text and figures. Be consistent.

*No changes were made. We consistently use "Nanog" to designate the human or mouse protein. We use "NANOG" only in the context of a specific motif or experiment from another resource (HOCOMOCO, ReMap, TFClass), and only if it is linked by this resource to the human NANOG gene. (Note that the gene symbols for the human and mouse Nanog proteins are NANOG and Nanog, respectively.)*

Both "in vivo" and "in vivo" appear in the text. Be consistent.

*Done .We consistently use italics in the main text. There is one non-italicized "in vivo" in the Reference Section, which we imported automatically from Pubmed.*

P. 27, l. 37: i is not italicized in "in vitro".

*Done, we also corrected an incompletely italicized "in vivo".*

P. 29, l. 22: add a comma after Thus.

*Done.*

**Third round of review**

**Reviewer 1**

The manuscript has improved significantly. We only have minor concerns this time.

Minor comments

1.     Background: add "(TF)" after first appearance of transcription factor.
2.     Figure 1, caption: "TFBS" comes after transcription factor, and not binding site.
3.     Background: Remove comma from "HT-SELEX, and PBM".
4.     We recommend considering using an abbreviation for binding site as BS.
5.     Background: Add comma after "computer with CentriMo (22)".
6.     There are a few unabbreviated "transcription factor"'s in the text. We recommend abbreviating them (except for the first occurrence under Background).
7.     Both hyphened and non-hyphened "DNA binding" appears in the text. Be consistent.
8.     Results, first paragraph: change comma after "images," to a dot.
9.     Results, first paragraph, add at the end "PBM data is publicly available via UniPROBE database (citation)".
10.     There's inconsistency in how A,B,C labels are denoted in Figure captions, i.e. parenthesis, dots, colons. Be consistent. We recommend parenthesis.
11.     The argument for the HT-SELEX 10% cut-off can be further strengthened by referring to Figure 1 in Pal et al. NAR 2019 Co-SELECT paper.
12.     Protocol for PBM data paragraph is not aligned as other paragraph (i.e. justified). This also applies to second paragraph on page 15.
13.     "10% and 50% percent" - percent is redundant.
14.     Results: "Zinc" is capitalized once - there should be consistency in its capitalization.
15.     Reference to Figure 5C is missing, should be "Figure 5 shows many … ChIP-seq (A), HT-SELEX 10% (B) and PBM (C)".
16.     Figures 5,6,S3 caption: "Only the TFs" should be "Only TFs".
17.     Figure 6 caption is not justified.
18.     Figure 5,6,S3: we recommend changing to "Pearson correlation" for consistency.
19.     "tenfold" -> "more than a tenfold".
20.     "log correlation" (several places in the text) -> "Pearson correlation", or does it mean something else? Log correlation is not defined in the text anywhere.
21.     "SELEX-data" -> "HT-SELEX data".
22.     Figure S4 - HT-SELEX experimental IDs are identical. Is it a mistake or was the negative set different between exp-1 and exp-2? This should be clarified or corrected.
23.     Figure S4 - a table should be presented as a table and not as a figure.
24.     Figure S4 - we recommend summarizing the results for all TFs with multiple experiments and PWMs. One example may be misleading.
25.     P.19, missing "and" before "factors with multiple".
26.     Figure 9, caption: missing "and" before "(C)".
27.     Table 1 and Figure S4 are the same display item, but one is a Table and one is a Figure. As stated before, we highly recommend converting a list of numbers to a table, and not a Unix-style table, but a proper formatted table.
28.     Table 1,2,3 captions are not justified.
29.     Table 1 caption - remove ")."
30.     Table 3 caption has some grammatic errors and is unclear, e.g. "TF's the family affiliation" and the last sentence with "?" and ambiguity.
31.     Figure S5, caption - "in vivo" is not italicized.
32.     P. 28, second sentence - remove second closing parenthesis.
33.     Figure S6 - please add correlation value and p-value for the correlation tests.
34.     Additional file 1: Description of additional files: should be justified and CISBP with a hyphen.
35.     "3'terminal" -> "3' terminal"
36.     Conclusions: Add commas to "software, such as the R environment, for ".
37.     Methods, p. 33: add a dot after "via a web interface ." and justify paragraph.
38.     Page 34: space missing after citation 29.

**Authors Response**

**Point-by-point responses to the reviewers’ comments:**

4.	We recommend considering using an abbreviation for binding site as BS.

*Not done. We feel that two many abbreviations would have a negative impact on the readability of the text.*

11.	The argument for the HT-SELEX 10% cut-off can be further strengthened by referring to Figure 1 in Pal et al. NAR 2019 Co-SELECT paper.

*We added the new reference to the paper.*

20.	"log correlation" (several places in the text) -> "Pearson correlation", or does it mean something else? Log correlation is not defined in the text anywhere.

*We consistently changed "log correlation" to "Pearson correlation". The "log" was meant to indicate that the correlation was computed between the log sum-occupancy scores and log probe intensities. However, since this is precisely described in the Methods Section, we agree that using the term "log correlation" at other places is unnecessary and potentially confusing.*

22.	Figure S4 - HT-SELEX experimental IDs are identical. Is it a mistake or was the negative set different between exp-1 and exp-2? This should be clarified or corrected.

*This was indeed a mistake. The experiment ID has now been corrected.*

23.	Figure S4 - a table should be presented as a table and not as a figure.

*Not done. Figure S4 contains two parts, one consisting of numbers that could be presented in a Table, plus a scatter plot. The two parts are thematically connected. Therefore, we prefer to keep them together in a single graphical item.*

27.	Table 1 and Figure S4 are the same display item, but one is a Table and one is a Figure. As stated before, we highly recommend converting a list of numbers to a table, and not a Unix-style table, but a proper formatted table.

*We prefer to leave this to the Production Editor, as we understand that the Table will be reformatted anyway and we are not sure whether an intervention from our side would be helpful.*

24.	Figure S4 - we recommend summarizing the results for all TFs with multiple experiments and PWMs. One example may be misleading.

*After consultation with the Editor, we decided not to follow up on this request, which seems to be something more like a major revision task. There are hundreds of TFs with multiple experiments and PWMs and we don't see an easy way to summarize the results in a transparent manner. Moreover, we consider the issue of cross-platform reproducibility outside the scope of this article. Finally, all benchmarking results are deposited in big data tables available from a public archive (zenodo) under a DOI identifier. Readers interested in the cross-platform reproducibility of binding assays for a particular TF can easily extract the corresponding performance measures from these table and carry out such an analysis in their own preferred way.*

30*.*	Table 3 caption has some grammatical errors and is unclear, e.g. "TF's the family affiliation" and the last sentence with "?" and ambiguity.

*We thank the reviewer for pointing this out. The Table legend has now been modified as follows:*

*"Only the 10 highest scoring experiments are shown. For HT-SELEX and PBM experiments, the TF's family affiliation as defined in TFClass, is shown in parenthesis: (1) POU domain, (2) NK-related (including NANOG), (3) paired-end, (4) HOX-related, (5) HD-LIM, (6) TCF-7-related, (7) SOX-related, (?) possibly outdated TF name (may in fact be Tcf7l1), (-) unclassified."*

*Background information: Many TFs are referred to by different names, and the same name is sometimes used do designate different TFs. In this particular case, the family of TCF-7-related TFs includes a member, whose official gene symbol is Tcf7l1 but which is often referred to by its synonym Tcf3. Unfortunately, Tcf3 is now the official gene symbol of a completely unrelated TF from the basic helix-loop-helix structural class. Therefore, we suspect that UniPROBE erroneously annotated the experiment with an outdated/unofficial TF name.*

33*.*	Figure S6 - please add correlation value and p-value for the correlation tests.

*An additional part (C) has been added to the Figure, which shows slopes and R^2^ values of trend lines between motif features and prediction performance measures. Numbers supported by significant correlation tests (P-value < 0.001) are presented in boldface.*

1*.*	Background: add "(TF)" after first appearance of transcription factor.

2.	Figure 1, caption: "TFBS" comes after transcription factor, and not binding site.

3.	Background: Remove comma from "HT-SELEX, and PBM".

5.	Background: Add comma after "computer with CentriMo (22)".

6.	There are a few unabbreviated "transcription factor"'s in the text. We recommend abbreviating them (except for the first occurrence under Background).

7.	Both hyphened and non-hyphened "DNA binding" appears in the text. Be consistent.

8.	Results, first paragraph: change comma after "images," to a dot.

9.	Results, first paragraph, add at the end "PBM data is publicly available via UniPROBE database (citation)".

10.	There's inconsistency in how A,B,C labels are denoted in Figure captions, i.e. parenthesis, dots, colons. Be consistent. We recommend parenthesis.

12.	Protocol for PBM data paragraph is not aligned as other paragraph (i.e. justified). This also applies to second paragraph on page 15.

13.	"10% and 50% percent" - percent is redundant.

14.	Results: "Zinc" is capitalized once - there should be consistency in its capitalization.

15.	Reference to Figure 5C is missing, should be "Figure 5 shows many ? ChIP-seq (A), HT-SELEX 10% (B) and PBM (C)".

16.	Figures 5,6,S3 caption: "Only the TFs" should be "Only TFs".

17.	Figure 6 caption is not justified.

18.	Figure 5,6,S3: we recommend changing to "Pearson correlation" for consistency.

19.	"tenfold" -> "more than a tenfold".

21.	"SELEX-data" -> "HT-SELEX data".

25.	P.19, missing "and" before "factors with multiple".

26.	Figure 9, caption: missing "and" before "(C)".

28.	Table 1,2,3 captions are not justified.

29.	Table 1 caption - remove ")."

31.	Figure S5, caption - "in vivo" is not italicized.

32.	P. 28, second sentence - remove second closing parenthesis.

34.	Additional file 1: Description of additional files: should be justified and CISBP with a hyphen.

35.	"3'terminal" -> "3' terminal"

36.	Conclusions: Add commas to "software, such as the R environment, for".

37.	Methods, p. 33: add a dot after "via a web interface <link>." And justify paragraph.

38.	Page 34: space missing after citation 29.

*The other requests by the reviewer listed below concern purely typographical errors or inconsistencies. They all are corrected in the new version.*
